# A randomized controlled trial to evaluate outcomes with Aggrenox in patients with SARS-CoV-2 infection

**DOI:** 10.1371/journal.pone.0274243

**Published:** 2023-01-30

**Authors:** Amit Singla, Nicholas B. Dadario, Ashima Singla, Patricia Greenberg, Rachel Yan, Anil Nanda, Detlev Boison, Rakesh Malhotra, Sunil Patel, Suri Nipun, Kaur Maninderpal, Dorothy Castro, Sanaa Bdiiwi, Hala Boktor, Htay Htay Kyi, Anne Sutherland, Amee Patrawalla, Kevin Ly, Yingda Xie, Ashish Sonig, Priyank Khandelwal, James Liu, Joseph Koziol, Diana Finkle, Sara Subanna, Steven K. Libutti

**Affiliations:** 1 Department of Neurological Surgery, Rutgers New Jersey Medical School, Newark, New Jersey, United States of America; 2 Department of Neurological Surgery, Rutgers Robert Wood Johnson Medical School, New Brunswick, New Jersey, United States of America; 3 Department of OBGYN, Rutgers Robert Wood Johnson Medical School, New Brunswick, New Jersey, United States of America; 4 Biostatistics and Epidemiology Services Center (RUBIES), Rutgers School of Public Health, Rutgers University, Piscataway, New Jersey, United States of America; 5 Brain Health Institute, Rutgers University, Piscataway, New Jersey, United States of America; 6 Department of Medicine, Division of Nephrology, UCSD, San Diego, California, United States of America; 7 Department of Medicine, Rutgers New Jersey Medical School, Newark, New Jersey, United States of America; 8 Department of Neurological Surgery, Saint Barnabas Medical Center, Livingston, New Jersey, United States of America; 9 Department of Surgery, Rutgers Robert Wood Johnson Medical School, New Brunswick, New Jersey, United States of America; Public Library of Science, UNITED KINGDOM

## Abstract

**Background:**

Coronavirus disease 2019 (COVID-19) is an immunoinflammatory and hypercoagulable state that contributes to respiratory distress, multi-organ dysfunction, and mortality. Dipyridamole, by increasing extracellular adenosine, has been postulated to be protective for COVID-19 patients through its immunosuppressive, anti-inflammatory, anti-coagulant, vasodilatory, and anti-viral actions. Likewise, low-dose aspirin has also demonstrated protective effects for COVID-19 patients. This study evaluated the effect of these two drugs formulated together as *Aggrenox* in hospitalized COVID-19 patients.

**Methods:**

In an open-label, single site randomized controlled trial (RCT), hospitalized COVID-19 patients were assigned to adjunctive Aggrenox (Dipyridamole ER 200mg/ Aspirin 25mg orally/enterally) with standard of care treatment compared to standard of care treatment alone. Primary endpoint was illness severity according to changes on the eight-point COVID ordinal scale, with levels of 1 to 8 where higher scores represent worse illness. Secondary endpoints included all-cause mortality and respiratory failure. Outcomes were measured through days 14, 28, and/or hospital discharge.

**Results:**

From October 1, 2020 to April 30, 2021, a total of 98 patients, who had a median [IQR] age of 57 [47, 62] years and were 53.1% (n = 52) female, were randomized equally between study groups (n = 49 Aggrenox plus standard of care versus n = 49 standard of care alone). No clinically significant differences were found between those who received adjunctive Aggrenox and the control group in terms of illness severity (COVID ordinal scale) at days 14 and 28. The overall mortality through day 28 was 6.1% (3 patients, n = 49) in the Aggrenox group and 10.2% (5 patients, n = 49) in the control group (OR [95% CI]: 0.40 [0.04, 4.01], p = 0.44). Respiratory failure through day 28 occurred in 4 (8.3%, n = 48) patients in the Aggrenox group and 7 (14.6%, n = 48) patients in the standard of care group (OR [95% CI]: 0.21 [0.02, 2.56], p = 0.22). A larger decrease in the platelet count and blood glucose levels, and larger increase in creatinine and sodium levels within the first 7 days of hospital admission were each independent predictors of 28-day mortality (p < 0.05).

**Conclusion:**

In this study of hospitalized patients with COVID-19, while the outcomes of COVID illness severity, odds of mortality, and chance of respiratory failure were better in the Aggrenox group compared to standard of care alone, the data did not reach statistical significance to support the standard use of adjuvant Aggrenox in such patients.

## 1 Introduction

A novel coronavirus was first reported in December 2019 and declared an international pandemic by the World Health Organization (WHO) on March 11, 2020. Approximately, 18–19 months later, more than 250 million cases have been confirmed resulting in over 5 million deaths [1]. A number of new therapeutic strategies have been examined globally to attempt to mitigate disease severity and overall mortality related to this virus, yet there remains limited FDA authorized medications to treat coronavirus disease 2019 (COVID-19) patients with generally equivocal results, such as Remdesivir, Baricitinib, Molnupiravir, and Paxlovid [2–6]. Thus, further development of effective preventative and therapeutic interventions to combat this disease is paramount as we continue to move forward in the ongoing pandemic and as new variant strains are identified.

COVID-19 is caused by severe acute respiratory syndrome coronavirus 2 (SARS-CoV-2), a single-stranded, positive-sense RNA virus belonging to a group of *Beta-coronaviruses* of the *Coronaviridae* family [7]. Recent data suggests the role of an exaggerated immunoinflammatory response and hypercoagulability as pathogenic mechanisms of COVID-19 responsible for clinical manifestations such as acute respiratory distress syndrome (ARDS), stroke, and extrapulmonary multiorgan dysfunction [8]. A few retrospective and review studies suggest a major risk of thromboembolic complications in COVID-19 patients, causing many to converge on the hypothesis that general endothelial dysfunction and activation of the coagulation system disrupts immune, renin-angiotensin-aldosterone, and thrombotic balance [9–11]. In response, the International Society of Thrombosis and Hemostasis has recommended that all COVID-19 patients who are admitted to the hospital, irrespective of the severity of their illness, should be started on prophylactic dose of low molecular weight heparin (LMWH) [12]. The Society of Thrombosis and Haemostasis also recommended that all hospitalized COVID-19 patients should receive pharmacological venous thromboembolism (VTE) prophylaxis unless contraindicated [13]. However, studies on treatment with anticoagulant agents continue to produce mixed results [14–16].

Antiplatelet therapy has provided another avenue of great interest to reduce platelet adhesion and aggregation, which is a major consequence of COVID-19 induced renin-angiotension system (RAS) activation [10]. Aspirin, a widely available therapy, demonstrates both anti-platelet and anti-inflammatory properties and has been shown to reduce in-hospital mortality in COVID-19 patients in a number of observational studies [17, 18], however such favorable results with Aspirin were not reflected in the recently conducted large RECOVERY randomized control trial in 2021 [19].

Utilization of a therapy that can simultaneously intervene along multiple pathways (i.e: viral replication, inflammation, vasoconstriction, and coagulation) may provide an important mechanism to improve patient outcomes. One particular agent, *Dipyridamole* (DIP), has recently emerged as a potential therapeutic agent for COVID-19 patients due to its broad spectrum antiviral activity against positive stranded RNA viruses and VSV-induced viral pneumonia models in vivo [20, 21], its immunomodulatory activity through inhibition of adenosine uptake into the endothelial and other cells [22] and its phosphodiesterase (PDE) inhibitor activity that may reduce the progressive tissue fibrosis in organs targeted by COVID-19 [23]. Of note, a recent screen of FDA approved drugs identified the ability of DIP to effectively suppress SARS-CoV-2 replication with a therapeutically achievable EC50 concentration of 100 nM [24]. Moreover, a small study indicated adjunctive therapy with DIP in 14 COVID-19 patients suggested improved outcomes including a reduction in coagulation markers [21].

Thus, we hypothesized that *Aggrenox* (Dipyridamole ER 200mg/ Aspirin 25mg orally/enterally) adjunctive therapy would be useful for SARS-CoV-2 infected patients by combining the anti-inflammatory and anti-thrombotic properties of DIP and aspirin exerted by different mechanisms (adenosine augmentation versus inhibition of cyclooxygenase). In a single center, open-label, prospective, randomized controlled trial (RCT), this study investigated the efficacy of Aggrenox in hospitalized patients with COVID-19 by examining both the raw values and the change in illness severity (using the COVID ordinal scale), all-cause mortality, and occurrence of respiratory failure.

## 2 Methods

### 2.1 Study design and participants

This study was a two-arm, open-label, single site randomized controlled trial (RCT) conducted in an urban academic medical center in New Jersey, United States of America. FDA-approval was obtained for exemption from Investigational New Drug Application (IND) to conduct a clinical investigation with the drug Aggrenox (Dipyridamole ER 200mg/ Aspirin 25mg). A placebo group was not included in the current study given the ethical concerns of the authors for not providing treatment for COVID-19 hospitalized patients especially when FDA approved drugs existed at the time of study [2, 25]. The trial protocol was approved by our center’s institutional review board (ClinicalTrials.gov Identifier: NCT04410328). The trial was overseen by a data-safety monitoring board of independent experts.

All the patients included in this trial were admitted to the hospital during the 2^nd^ wave of COVID-19 in the northeastern United States (between October 1, 2020 to April 30, 2021). This study was completed prior to the widespread availability of vaccines used under emergency use authorization, and therefore no study patients were vaccinated at any point during the study evaluation period. All patients, or their approved health care proxies or next of kin, provided written informed consent prior to study enrollment.

Adult patients (aged ≥18 years) who were hospitalized for suspected symptomatic SARS-CoV-2 infection with a high clinical suspicion for COVID-19 (fever and cough for ≤ 7 days, bilateral pulmonary infiltrates on imaging or new hypoxemia with spO2 ≤94% on room air or no alternative explanation for respiratory symptoms) were eligible for inclusion in the study. Patients required a positive laboratory test for SARS-CoV-2 infection within 3 days of hospitalization. Patients were then excluded based on the following criteria: pregnancy; history of G6PD deficiency; antiplatelet agents including inhibitor of P2Y12 ADP platelet receptors, phosphodiesterase inhibitors, and glycoprotein IIB/IIIA inhibitors; therapeutic anticoagulation with coumadin, unfractionated or low molecular weight heparin, and direct oral anticoagulants; vasodilatory shock; known ongoing angina, recent myocardial infarction and sub-valvular aortic stenosis; active gastric or duodenal ulcer or any bleeding disorder or creatinine clearance < 10; hemoglobin <9 mg/dL, platelet count of <30,000 /mm^3^; acute respiratory infection for >10 days; known allergy/hypersensitivity to dipyridamole and/or aspirin; severe hepatic or renal insufficiency; uncontrolled hypertension defined as systolic > 180 mm Hg or diastolic > 100 mm Hg; patients with known allergy to NSAIDs; patients enrolled in other non-standard of care clinical trials.

#### Randomization and masking

Participants were randomly assigned to receive either adjunctive Aggrenox treatment along with standard of care (SOC) (Aggrenox group) or SOC (control group) alone in a 1:1 ratio using random permuted blocks. The randomization sequence was concealed from study staff and was integrated into the REDCap electronic data capture system built to record the study’s patient data by the study biostatistician. Patients were assessed daily while hospitalized and outcomes were examined through days 14, 28, and/or discharge. A COVID-19 baseline electronic case report form (eCRF; as available on the WHO website for COVID-related studies) was completed at admission and the daily core CRF were completed on subsequent days for the duration of hospitalization. Patients and health care providers were not masked to study group assignment.

#### Procedures

Patients in the Aggrenox group received Aggrenox (dipyridamole ER 200mg and aspirin 25mg), 2 times daily for a total of 2 weeks along with the SOC treatment. Standard of care treatment consisted of an intravenous Remidesivir 200 mg loading dose and then 100 mg daily for a total of 4 days for non-intubated patients and 10 days for intubated patients, intravenous/oral decadron 6 mg daily for 10 days and prophylactic subcutaneous LMWH daily, started on the day of enrollment and for the duration of hospitalization. If the patients were discharged before 10 days, they were prescribed oral decadron 6 mg daily to complete the course of 10 days. Patients in the control group only received SOC treatment as described above. No patients enrolled in this study received other experimental antivirals or immunomodulators used for COVID-19. Thus, regardless of randomization, all patients were treated according to the SOC protocol at the time of the study for COVID-19 according to the state of New Jersey.

Follow-up visits were completed through phone-calls on day 14^th^ (+2 days) and day 28^th^ (+2 days) to assess continued dependency on oxygen therapy, activity limitations, shortness of breath, and/or death outside of the hospital.

#### Outcomes

The primary study outcome was change in composite COVID-ordinal scale (S1 Table in S1 File) from baseline, compared between study groups. Evaluation on scale was made from baseline (day 1) to days 14, 28, and/or hospital discharge. The scale used in this study consisted of 8 levels: 1) not hospitalized with resumption of normal activities; 2)not hospitalized, but unable to resume normal activities; 3) hospitalized, not requiring oxygen; 4) hospitalized, requiring oxygen; 5) hospitalized, requiring high-flow oxygen therapy, or noninvasive ventilation; 6) hospitalized, requiring invasive ventilation; 7) ventilation plus additional organ support such as vasopressors, renal replacement therapy and ECMO; and 8) death.

The secondary study outcomes included all-cause mortality and/or respiratory failure through days 14, 28, or final follow-up, compared between the study groups. Respiratory failure was defined as those requiring high-flow oxygen therapy, noninvasive ventilation, invasive ventilation, and/or death (levels 5–8 on COVID ordinal scale) per prior published studies [26]. COVID-19 related complications, adverse, and serious adverse events (SAEs) related to the study drug data were also collected. The causality of adverse and SAEs (their relationship to all study treatment/procedures) was assessed by the study investigator(s) and was communicated to the study sponsor via an SAE form and the FDA via MedWatch form, if applicable.

Laboratory markers including hemoglobin, WBC, lymphocytes, neutrophils, hematocrit, platelets, ALT/SGPT, bilirubin, AST/SGOT, glucose, BUN, creatinine, sodium, potassium, CRP, d-dimer, ferritin, lactate dehydrogenase, and other independent risk factors (CPD, diabetes, CVD, HTN, Aids/HIV, Smoking status) were also assessed and compared between study groups.

### 2.2 Statistical analysis

A sample size of 132 patients (66 patients per arm) was estimated to provide 90% power, while a sample size of 100 patients (50 patients per arm) was estimated to provide 80% power, to detect a relative between-group difference of 20% reduction in composite ordinal scale (from baseline to day 14) with an assumption of a mean ± standard deviation composite ordinal scale value of 4.5 ± 1.6 at baseline and two-sided alpha value of 0.05.

All baseline patient demographics, presenting symptomology, comorbidities, recent medication usage, and clinical outcome risk factors were summarized using range, mean and standard deviation (SD), and median with interquartile range (IQR) for continuous measures, or as frequencies with percentages for categorical measures. In order to compare these measures between the two treatment groups, Pearson Chi-Square and Fisher Exact tests were used for categorical measures, while independent student t-tests were used for normally distributed continuous measures, and Wilcoxon Rank Sum tests were used for ordinal and non-normally distributed continuous measures. Normality was assessed using Shapiro-Wilk and Kolmogorov–Smirnov testing along with visual inspection of quantile-quantile plots. Patient discharge measures, laboratory values, and primary and secondary outcomes were analyzed in the same way prior to any multivariable analyses.

To further assess the primary and secondary outcomes, multivariable regression models were used. In order to examine the change in the composite COVID ordinal scale from baseline to day 14 and day 28, multivariable linear regression was used. For the patients’ raw COVID ordinal scale measure at day 14 and day 28, ordinal multivariable logistic regression was used. However, due to the small number of patients who belonged to most of the levels at both time points, the proportional odds assumption was violated. Therefore, these models were built using an unequal slopes assumption (for non-proportional odds), and results were summarized as odds ratios with 95% confidence by cumulative scale level. Additionally, due to low event rates, multivariable logistic regression with Firth’s penalized likelihood was used to model both all-cause mortality and respiratory failure at days 14 and 28, and results were summarized as odds ratios with 95% confidence intervals [27].

In addition, Kaplan Meier curves were used to plot all-cause mortality at day 14, day 28, and overall. Log Rank tests were used to determine whether there were significant differences between the curves for the two treatment groups. All statistical analyses were completed using SAS software version 9.4 (SAS Institute Inc., Cary, NC, USA) and R version 4.1.1 (R Foundation for Statistical Computing, Vienna, Austria), where a two-sided p-value < 0.05 was considered statistically significant.

## 3 Results

### 3.1 Demographics and presenting symptoms

**Fig 1:**
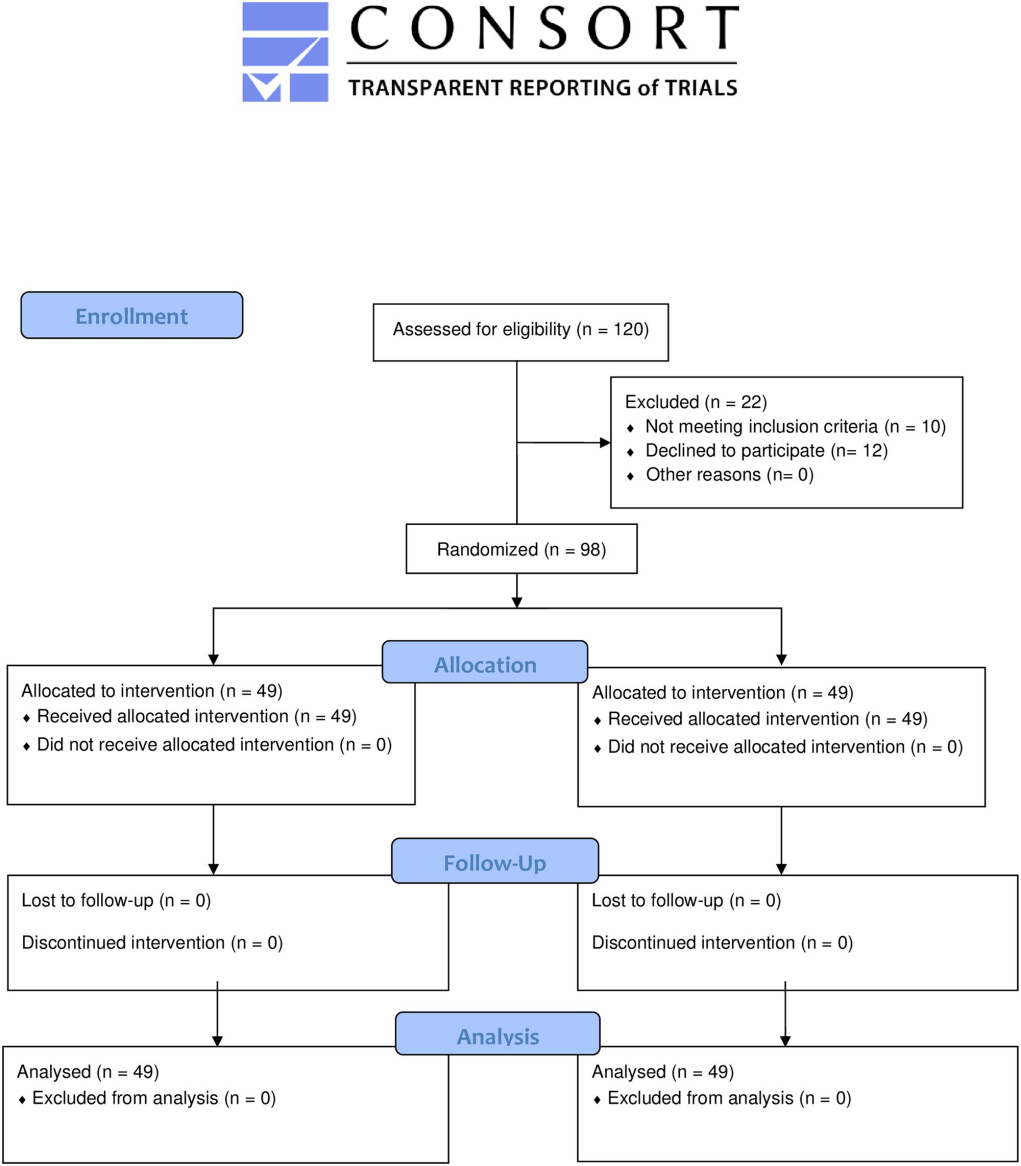
Consort 2010 flow diagram.

From October 1, 2020 to April 30, 2021, a total of 98 patients with RT-PCR confirmed COVID-19 were enrolled and randomized equally between study groups (n = 49 per group). See consort flow diagram for details on enrollment and analysis (Fig 1). Patient demographics were similarly represented between study groups as shown in S1 Table in S1 File. The median [IQR] age of enrolled patients was 57 years [47, 62], and the patient sample consisted of 53.1% females (n = 52), and had a median [IQR] BMI equal to 29.4 [25.8, 35.5]. Sex was the only statistically significant demographic difference between study groups, in which there were more females in the Aggrenox group (63.3%) compared to the control group (42.9%), p = 0.04. On average, patients in full study sample presented with symptoms starting approx. 5 days before admission, with a history of shortness of breath (80.6%), cough (79.6%), and fever (77.5%). Non-constitutional symptoms mostly consisted of chest pain (29.6%) and altered consciousness or confusion (6.1%). A list of additional symptoms recorded is shown in S2 Table in S1 File. Pre-hospital medications were well represented between groups, with most common medications being NSAIDs (Aggrenox group: 15 (30.6%) vs control: 14 (28.6%); p = 0.97) and Angiotensin-Converting Enzyme (ACE) Inhibitors (Aggrenox group: 4 (8.2%) vs control: 9 (18.4%); p = 0.37). Non-significant differences in antibiotic, antivirals, oral steroid, and angiotensin II receptor blocker (ARB) use at hospital admission are shown in S3 Table in S1 File.

### 3.2 Primary outcome

Based on univariate analyses, both groups demonstrated statistically similar mean (SD) changes on the 8-point COVID ordinal scale of increasing illness severity through day 14 (Aggrenox: 2.3 (1.8) vs control: 2.1 (2.0); p = 0.74) and day 28 (Aggrenox: 2.5 (1.7) vs control: 2.0 (2.2), p = 0.24). More specifically, the majority of patients (82.7%) in both study groups improved from days 1 to 14 (Aggrenox: 41 vs control: 40), more patients in the control group got worse (Aggrenox: 4 vs control: 6), and all other patients showed no change (n = 7, 7.1%). Similar trends were demonstrated when analyzing data through day 28, where 85.7% of patients improved from baseline (Aggrenox: 44 vs control: 40) and 10.2% of patients got worse (Aggrenox: 4 vs control: 6), while 2 patients (4.1%) in the control showed no change and 1 patient in each group (2.0%) was removed due to missing data. These data are presented in S2 Table in S1 File.

After controlling for other potential risk factors including: comorbidities (CPD, diabetes, CVD, HTN, Aids/HIV, Smoking status), DBP at baseline, recent use of NSAIDS and oral steroids, and baseline lab values (hemoglobin, WBC, lymphocytes, neutrophils, hematocrit, platelets, ALT/SGPT, bilirubin, AST/SGOT, glucose, BUN, creatinine, sodium, potassium, CRP, d-dimer, and ferritin, lactate dehydrogenase) there was no significant difference in the change in the composite COVID ordinal scale from baseline to day 14 and day 28 based on the treatment group (p = 0.32 and p = 0.31, respectively). After removing the lab value predictors which caused a large drop in sample size due to missing data, treatment group was still not a significant predictor of change in the composite COVID ordinal scale from baseline to day 14 (p = 0.23). However, for the day 28 linear regression model without lab values, treatment group was marginally significant in the full model (β = 0.86, p = 0.07) (S4 Table in S1 File).

Again, the multivariable ordinal logistic models for patients’ raw COVID ordinal scale measure at days 14 and 28 showed no significant differences between the treatment groups for all cumulative COVID scale levels (all p = 1.0); however, the models failed to fully converge even after allowing for uneven slopes (non-proportional odds). After applying both an uneven slopes assumption and stepwise selection methods to pair the model down to only treatment group, plus any remaining significant factors, only treatment group remained in both the day 14 and day 28 outcome models. When comparing those who received Aggrenox versus the control group in terms of illness severity at day 14, there were no significant differences for those who recovered and could resume normal activities versus everyone else (OR [95% CI]: 0.46 [0.14, 1.43], p = 0.18); those who were discharged versus those who were not (OR [95% CI]: 0.33 [0.08, 1.32], p = 0.12); those who were discharged or were still in the hospital without requiring oxygen versus those who required therapies or died (OR [95% CI]: 0.29 [0.06, 1.49], p = 0.14); those who were discharged or were still in hospital with or without oxygen only versus those who required more involved therapies or died (OR [95% CI]: 0.21 [0.02, 1.87], p = 0.16); those who were discharged or were still in hospital with or without only noninvasive therapy versus those who required invasive therapies or died (OR not estimable, p = 0.99); those who were discharged or were still in hospital with or without pulmonary therapy only versus those who required additional organ support or died (OR not estimable, p = 1.0). However, when comparing all surviving patients versus those who died, there was a significant difference (OR [95% CI]: 11.75 [1.29, 107.10], p = 0.03), suggesting that patients on Aggrenox have significantly higher odds of being alive versus dead on day 14. No significant differences were found between those who received Aggrenox and the control group in in terms of illness severity at day 28 (OR [95% CI]: 1.62 [0.52, 4.96], p = 0.40; 1.88 [0.51, 6.89], p = 0.34; 1.74 [0.39, 7.75], p = 0.47) when comparing those who recovered and could resume normal activities versus everyone else, those who were discharged versus those who were not, and all surviving patients versus those who died, respectively.

### 3.3 Secondary outcomes

**Fig 2:**
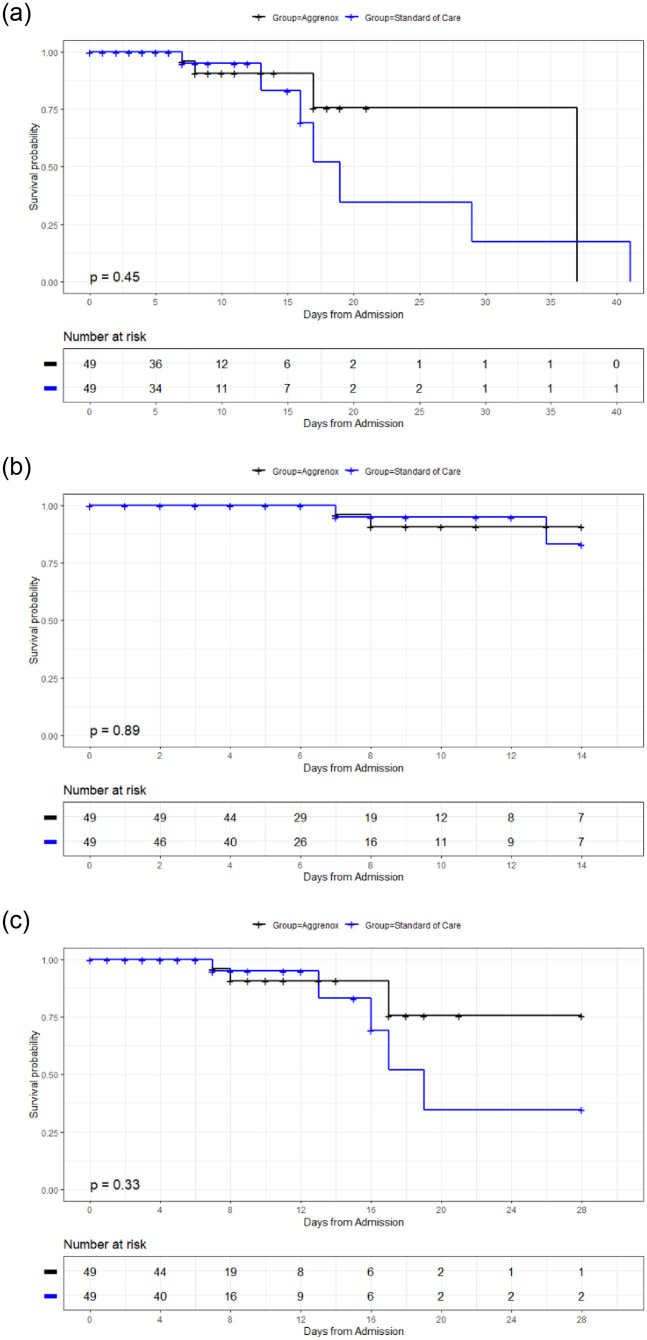
Overall K-M Mortality (a), day 14 (b) and day 28 (c).

The overall mortality rate was 11.2% (n = 11). Univariate analyses suggested non-significant differences between study groups for the outcome of all-cause mortality, although less patients in the Aggrenox group died compared to the control group (Aggrenox: 4 vs control: 7; p = 0.34). Furthermore, while not statistically significant, Kaplan-Meier analyses visually suggest an increasing trend of later death in the control group as day 28 was approached (Fig 2a–2c). When controlling for possible risk factors including age, sex, obesity status, and comorbidities (CVD, CPD, diabetes, smoking status), multivariate analyses demonstrated non-significant differences between the Aggrenox and control groups on the outcome of mortality through day 14 (OR [95% CI]: 0.85 [0.11, 6.31], p = 0.87) and through day 28 (OR [95% CI]: 0.40 [0.04,4.01], p = 0.44).

A total of 11 (11.2%) patients experienced respiratory failure, defined as those requiring high-flow oxygen therapy, noninvasive ventilation, invasive ventilation, and/or death (levels 5–8 on COVID ordinal scale). Most of the patients who experienced respiratory failure were in the control group (Aggrenox: 4 vs control: 7), but these data failed to reach statistical significance on univariate analyses (p = 0.34). When controlling for possible risk factors including age, sex, obesity status, and comorbidities (CVD, CPD, diabetes, smoking status), multivariate analyses demonstrated non-significant differences between Aggrenox and the control group on the outcome of respiratory failure through day 14 (OR [95% CI]: 0.49 [0.07, 3.51], p = 0.48) and through day 28 (OR [95% CI]: 0.21 [0.02, 2.57], p = 0.22). Multivariate regressions for mortality and respiratory failure are shown in S3 Table in S1 File.

### 3.4 COVID-19 related complications

The median [IQR] duration of hospital stay was 6 [4, 9] days, which was similar between groups (p = 0.45). A total of 12 patients were admitted to the ICU or high dependency unit (Aggrenox: 5 vs control: 7, p = 0.88), which lasted a median of 2 [1, 5] days. All patients required some form of oxygen therapy, which lasted a median of 5 [4, 9] days and was similarly represented between study groups (p = 0.82). The oxygen therapy mostly consisted of non-invasive ventilation in 26.5% of patients (Aggrenox: 12 vs control: 14, p = 0.15) compared to invasive ventilation in 9.2% of patients (Aggrenox: 4 vs control: 5, p = 1.00). All other patients (64.3%) received oxygen therapy with nasal cannula only. The maximum oxygen flow volume received in patients was statistically similar between study groups (p = 0.36).

The most common complications included liver dysfunction among 21.4% of patients (Aggrenox: 9 vs control: 12, p = 0.54) and acute renal injury among 14.3% of patients (Aggrenox: 5 vs control: 9, p = 0.71). Disseminated intravascular coagulation occurred in only 3 patients, all of which were in the control group (6.1%). Additional antibiotics were required in a total of 17.3% of patients (Aggrenox: 5 vs control: 12, p = 0.14). These data are presented in S4 Table in S1 File, with an extended list of COVID-19 related complications shown in the S5 Table in S1 File.

### 3.5 Study drug-related adverse and SAEs

No adverse or serious adverse events secondary to the study drug were seen in patients in the experimental treatment group.

### 3.6 Inflammatory markers for illness severity

Inflammatory markers at patient presentation (day 1) which correlated with increased mortality through day 28 include decreased lymphocytes and platelets, as well as increased D-dimer, creatinine, C-reactive protein (CRP), urea, troponin, potassium, lactate dehydrogenase (LDH), and blood glucose levels. Patients that had a higher risk of 28-day mortality had a greater magnitude of change from day 1 to day 7 in decreased platelets, decreased blood glucose, increased creatinine, and increased sodium. Further values are represented in the S6 Table in S1 File.

Inflammatory markers at patient admission (day 1) which correlated with increased respiratory failure through day 28 included: decreased lymphocytes and platelets, as well as increased D-dimer, white blood cell counts, blood glucose, urea, lactate, creatinine, procalcitonin, CRP, LDH, and troponin. Patients that had a higher risk of 28-day respiratory failure had a greater magnitude of change from day 1 to day 7 in decreased platelets, increased total bilirubin, and increased potassium levels. Further values are represented in the S7 Table in S1 File.

There were no statistically significant differences in any inflammatory markers between the Aggrenox and control groups on univariate analyses (S8 Table in S1 File). Similarly, multivariate analyses identified no statistically significant inflammatory markers associated with the incidence of mortality through day 14 or day 28. Furthermore, our multivariate analyses revealed no statistically significant inflammatory markers associated with the incidence of respiratory failure through day 14 or day 28.

## 4 Discussion

In this RCT involving 98 hospitalized patients with confirmed COVID-19, an FDA-approved standard therapeutic-dose of Aggrenox in addition to the SOC treatment did not result in significantly reduced overall illness severity on the COVID ordinal scale, all-cause mortality, or respiratory failure as compared to the patients only receiving SOC treatment. Our results refute our hypothesis that use of Aggrenox as an adjunctive therapy improves outcomes for hospitalized SARS-CoV-2 infected patients at standard dose compared with SOC treatment alone. However, as fewer instances of these outcomes occurred in patients taking Aggrenox, additional studies utilizing adenosine augmenting therapies appear warranted in the treatment of COVID-19 moving forward.

Our overall hypothesis is primarily based on the scientific premise that dipyridamole (DIP) increases extracellular adenosine, a potent immunoregulatory nucleoside that interferes with inflammatory processes by modulating the biosynthesis and release of proinflammatory cytokines, slowing down the oxidative activity, preventing platelet aggregation, and reducing neutrophils adhesion and degranulation. Specifically, DIP blocks intracellular uptake of extracellularly- generated adenosine thereby increasing extracellular adenosine. This results in the up-regulation of adenosine A2A receptors (A2AR) signaling which exerts anti-inflammatory and anti-thrombotic effects through cAMP [28]. Furthermore, COVID-19 presents an exaggerated immunoinflammatory response and hypercoagulable state due to mechanisms including RAS activation and general endothelial damage. Therefore, a multi-functional drug such as Aggrenox, which combines the anti-inflammatory and antiplatelet properties of DIP and aspirin, was expected to be beneficial in the setting of COVID-19, by synergistically targeting several pathways involved in the regulation of inflammatory and vascular properties.

COVID-19 induces venous and arterial micro- and macrothrombotic events resulting in complications such as venous thromboembolism, peripheral arterial disease and stroke [9, 10]. These reports prompted a search for better thrombosis prevention, and anticoagulatory therapies have quickly become encouraged to be utilized in hospital settings to attempt to reduce COVID-19 related complications, despite a lack of evidence from ongoing RCTs [15, 16] A number of observational studies have suggested that therapeutic-dose anticoagulation improves COVID-19 outcomes [11, 18, 29], however conflicting results have been produced in recent RCTs examining enoxaparin [16] and heparin [15]. Antiplatelet drugs such as aspirin have been assessed by a number of retrospective studies as well [17, 18] suggesting in-hospital aspirin reduces the incidence of in-hospital mortality. However, recent results from the RECOVERY RCT of 2,521 patients suggested aspirin was not associated with a reduction in 28-day mortality nor progression to invasive mechanical ventilation or death [19]. Antiplatelet drugs other than aspirin such as ticagrelor [30] and dipyridamole [31] have been suggested as a potential therapeutic as well to be evaluated in a randomized clinical trial in COVID-19. However, the evidence for the use of antiplatelet drugs other than aspirin in a RCT setting is lacking.

ATTAC-19 is the first RCT to investigate the role of Aggrenox in improving patient outcomes after COVID-19. Our results showed that in patients hospitalized with COVID-19, Aggrenox was not associated with statistically significant reductions in 28-day mortality or in the risk of progressing to invasive mechanical ventilation or death. Therefore, results from the current trial do not justify the additional use of Aggrenox on top of SOC practice for hospitalized COVID-19 patients.

A number of potential hypotheses may explain our results. First, despite the controversial results demonstrated in the recent literature on in-hospital aspirin, earlier use of anti-inflammatory and anti-platelet drugs, such as Aggrenox at the beginning of symptoms may be more beneficial for moderately to critically ill patients. Preexisting aspirin prescription among COVID-19-positive veterans was found to result in a statistically and clinically significant decreases in overall mortality at 14-days (OR [95% CI]: 0.38 [0.32,0.46] and at 30-days (OR [95% CI]: 0.38 [0.33, 0.45]) [32]. It is possible that once COVID-19 disease has already progressed, in-hospital use of antiplatelets may not reduce the propagation of neutrophil recruitment in pulmonary endothelium and thrombus formation [33].

Secondly, although DIP has demonstrated the ability to indirectly reduce many of the pathogenic mechanisms of COVID-19 in other settings, such as influenza [34], herpes simplex virus [35], respiratory tract infections [36], and even other positive-stranded RNA viruses [20], all of these diseases have uniquely different disease processes than the largely still unknown COVID-19 disease. Only one study has been published suggesting DIP has a potential to benefit COVID-19 patients [21], however that study reported the outcomes in only a small number of patients recruited in a non-randomized fashion. Lastly, even though the combination of low-dose aspirin with DIP together has been suggested to be twice as effective as either aspirin or DIP alone [37], that may not necessarily synergistically attenuate the exaggerated immune response seen in COVID-19 patients, especially if administered when the disease has already progressed.

Currently, improving vaccination rates remains the most effective method of preventing widespread viral dissemination and global morbidity and mortality due to COVID-19 infection. However, emerging data demonstrates waning immunity just 6 months after vaccination, illustrating the importance of continuing to identify prognostic markers of disease severity for earlier interventions and resource allocation as well as further examination of possible drug therapies [38]. We identified a number of inflammatory markers that correlated with death and/or respiratory failure that could serve as markers for risk stratification. Increased D-dimer, CRP, and lymphocytopenia have correlated with worse outcomes in prior reports, as well as our study [39]. Further risk markers identified for illness severity and risk of death include increased urea, lactate dehydrogenase, and troponin levels, as well as decreased platelets and potassium levels. Interestingly, in addition to patients with greater changes in creatinine and platelet levels from day 1 to day 7, patients with greater decreases in glucose levels from day 1 to day 7 had an increased risk of 28-day mortality and respiratory failure. Identification of high blood glucose as a proxy for disease progression and severity has been only recently suggested, with evidence to support its participation in multiple mechanisms that accelerate nearly each step of the SARS-CoV-2 life cycle [40]. Our results add to this limited literature and suggest future study is necessary to examine the protective effect of blood glucose control and avoiding high levels of IV glucose in high dependency units on illness severity.

### 4.1 Limitations

The ATTAC-19 trial is not without its limitations. It is important to mention the current trial did identify a decrease in respiratory failure and all-cause mortality for the Aggrenox group compared to SOC alone, despite these data not reaching statistical significance. For instance, Aggrenox resulted in nearly half the number of deaths compared to the control group (n = 4 vs 7). Unfortunately, one patient could not be contacted in the Aggrenox group despite repeated efforts. It is possible that with additional patients, this difference would become statistically significant. The sample size for the current study was acquired with the goal to detect a relative between-group difference of 20% reduction on the COVID-19 ordinal scale discussed above. Twenty percent reduction was chosen to determine the sample size as we included only hospitalized patients ranging from COVID-ordinal scale 4 to 7 and we considered that a 1-point change in patient’s COVID-ordinal scale would be a significant improvement in hospitalized patient’s clinical status, such as improving from invasive ventilation to non-invasive ventilation. Previous larger studies have varied in their methods to plan an appropriate sample size which could detect a meaningful intervention effect, and there has been a lack of standard methods across trials especially given the lack of historical data for COVID-19 treatments which could inform these analyses. Other trials also examining ordinal scale outcomes have include larger percent reductions between groups (eg, 40%) [41], differences in odds ratio ranging between 0.5–1.8 [2, 42], and even no sample size calculation due to a lack of preexisting data on effect size for a novel COVID-19 treatment [43]. In the absence of a reliable pilot study or historical dataset for the current treatment being investigated, conventional sample size estimations with parametric or non-parametric analyses according to assumptions of sample distribution is appropriate as utilized in the current work [44].

The current study was an open-label, single site design and thus is inherently subject to ascertainment bias on the outcome of respiratory failure and also a possible selection bias. While we believe that it was clinically evident which patients indeed required high flow oxygen or some form of non-invasive/invasive ventilation therapy, and therefore did not likely interfere with our results, it is possible that our sample represented a different group of patients compared to that of the general population. Other studies have reported much higher mortality rates during similar study periods [45], and therefore Aggrenox could have resulted in larger benefits or complications in other patient samples. Lastly, our study predated the introduction of modern vaccines which are now globally available, and therefore it is unclear how Aggrenox may work in patients with previous antibodies, as is for most trials for our study period.

## 5 Conclusion

Adjuvant Aggrenox therapy was not associated with significantly reduced illness severity, respiratory failure, or all-cause mortality in hospitalized COVID-19 patients as compared with standard of care treatment alone. While these outcomes were numerically reduced in patients on both Aggrenox and SOC treatment, there is currently insufficient evidence to justify the standard use of adjuvant Aggrenox treatment in hospitalized patients with COVID-19.

## Supporting information

S1 File (DOCX)

S2 File (PDF)
