# Supplementary material for: A randomized controlled trial to evaluate outcomes with Aggrenox in patients with SARS-CoV-2 infection
Source: PLoS One. 2023 Jan 30;18(1):e0274243. doi: 10.1371/journal.pone.0274243 (PMC9886260; doi:10.1371/journal.pone.0274243)
Supplement: S1 File — (DOCX) [file pone.0274243.s001.docx]

**A randomized controlled trial to evaluate outcomes with Aggrenox in patients with SARS-CoV-2 infection**

**Supplementary Material**

Trial Registration: ClinicalTrials.gov Identifier: NCT04410328

**COVID Ordinal Scale:**

| 1 | not hospitalized with resumption of normal activities |
| --- | --- |
| 2 | not hospitalized, but unable to resume normal activities |
| 3 | hospitalized, not requiring oxygen |
| 4 | hospitalized, requiring oxygen |
| 5 | hospitalized, requiring high-flow oxygen therapy, or noninvasive ventilation |
| 6 | hospitalized, requiring invasive ventilation |
| 7 | ventilation plus additional organ support such as vasopressors, renal replacement therapy and ECMO |
| 8 | death |

Supplementary Table S1. Covid Ordinal Scale.

| **SIGNS AND SYMPTOMS AT HOSPITAL ADMISSION** | | | | | |
| --- | --- | --- | --- | --- | --- |
|  | **Total Study Sample (n = 98)** | **Aggrenox (n = 49)** | **Standard of Care (n = 49)** |  |  |
| **Time from Symptom Onset to Current Admission (days)** |  |  |  | WRS: p = 0.40 |  |
| Range | -11 - 14 | -11 - 14 | 0 - 14 |  |  |
| Mean (SD) | 5.3 (4.0) | 5.0 (4.4) | 5.7 (3.4) |  |  |
| Median [Q1, Q3] | 5 [3, 7] | 5 [2, 7] | 5 [3, 7] |  |  |
| Missing | n = 1 | n = 1 | n = 0 |  |  |
|  |  |  |  |  |  |
| **Temperature (deg F)** |  |  |  | WRS: p < 0.01 |  |
| Range | 93.7 - 103.2 | 93.7 - 103.0 | 97 - 103.2 |  |  |
| Mean (SD) | 99.8 (1.8) | 99.2 (1.8) | 100.4 (1.7) |  |  |
| Median [Q1, Q3] | 99.6 [98.5, 101.0] | 98.9 [98, 100.2] | 100.4 [98.8, 102.0] |  |  |
| Missing | n = 2 | n = 2 | n = 0 |  |  |
|  |  |  |  |  |  |
| **Heart Rate (bpm)** |  |  |  | WRS: p = 0.08 |  |
| Range | 67 - 139 | 67 - 136 | 68 - 139 |  |  |
| Mean (SD) | 97.4 (17.1) | 94.3 (17.7) | 100.5 (16.0) |  |  |
| Median [Q1, Q3] | 96 [86, 111] | 93 [78, 106] | 98 [89, 112] |  |  |
| Missing | n = 0 | n = 0 | n = 0 |  |  |
|  |  |  |  |  |  |
| **Respiratory Rate (bpm)** |  |  |  | WRS: p = 0.15 |  |
| Range | 16 - 40 | 16 - 40 | 16 - 40 |  |  |
| Mean (SD) | 20.7 (4.7) | 20.1 (4.4) | 21.3 (5.0) |  |  |
| Median [Q1, Q3] | 19 [18, 22] | 19 [18, 20] | 19 [18, 22] |  |  |
| Missing | n = 0 | n = 0 | n = 0 |  |  |
|  |  |  |  |  |  |
| **Systolic Blood Pressure (mmHg)** |  |  |  | WRS: p = 0.057 |  |
| Range | 87 - 207 | 87 - 156 | 101 - 207 |  |  |
| Mean (SD) | 131.1 (19.3) | 126.3 (15.6) | 135.9 (21.4) |  |  |
| Median [Q1, Q3] | 130 [120, 140] | 127 [116, 139] | 134 [121, 143] |  |  |
| Missing | n = 1 | n = 1 | n = 0 |  |  |
|  |  |  |  |  |  |
| **Diastolic Blood Pressure (mmHg)** |  |  |  | WRS: p < 0.01 |  |
| Range | 46 - 112 | 46 - 106 | 56 - 112 |  |  |
| Mean (SD) | 75.2 (12.3) | 71.5 (11.5) | 78.8 (12.0) |  |  |
| Median [Q1, Q3] | 73 [67, 83] | 69 [65, 79] | 77 [70, 89] |  |  |
| Missing | n = 1 | n = 1 | n = 0 |  |  |
|  |  |  |  |  |  |
| **Oxygen Saturation (%)** |  |  |  | WRS: p = 0.97 |  |
| Range | 50 - 99 | 50 - 99 | 50 - 98 |  |  |
| Mean (SD) | 89.9 (8.3) | 89.9 (8.7) | 90.0 (8.1) |  |  |
| Median [Q1, Q3] | 91 [88, 95] | 91 [88, 95] | 92 [88, 94] |  |  |
| Missing | n = 0 | n = 0 | n = 0 |  |  |
|  |  |  |  |  |  |
| **Sternal Capillary Refill Time >2 sec, n (%)** |  |  |  | FE: p = 0.53 |  |
| Yes (1) | 1 (1.0) | 0 (0.0) | 1 (2.0) |  |  |
| No (0) | 37 (37.8) | 17 (34.7) | 20 (40.8) |  |  |
| Unknown (2) | 60 (61.2) | 32 (65.3) | 28 (57.2) |  |  |
|  |  |  |  |  |  |
| **Obesity - based on BMI, n (%)** |  |  |  | CS: p = 0.57 |  |
| Yes: BMI = 30+ (1) | 41 (41.8) | 19 (38.8) | 22 (44.9) |  |  |
| No: BMI < 30 (0) | 45 (45.9) | 25 (51.0) | 20 (40.8) |  |  |
| Unknown (2) | 12 (12.2) | 5 (10.2) | 7 (12.3) |  |  |
|  |  |  |  |  |  |
| **History of Fever, n (%)** |  |  |  | FE: p = 0.08 |  |
| Yes (1) | 76 (77.5) | 34 (69.4) | 42 (85.7) |  |  |
| No (0) | 19 (19.4) | 12 (24.5) | 7 (14.3) |  |  |
| Unknown (2) | 3 (3.1) | 3 (6.1) | 0 (0.0) |  |  |
|  |  |  |  |  |  |
| **Cough, n (%)** |  |  |  | FE: p = 0.59 |  |
| Yes (1) | 78 (79.6) | 38 (77.6) | 40 (81.6) |  |  |
| No (0) | 18 (18.4) | 9 (18.4) | 9 (18.4) |  |  |
| Unknown (2) | 2 (2.0) | 2 (4.1) | 0 (0.0) |  |  |
|  |  |  |  |  |  |
| **Sore Throat, n (%)** |  |  |  | FE: p = 0.48 |  |
| Yes (1) | 15 (15.3) | 9 (18.4) | 6 (12.2) |  |  |
| No (0) | 77 (78.6) | 36 (73.5) | 41 (83.7) |  |  |
| Unknown (2) | 6 (6.1) | 4 (8.2) | 2 (4.1) |  |  |
|  |  |  |  |  |  |
| **Runny nose (rhinorrhea), n (%)** |  |  |  | FE: p = 0.25 |  |
| Yes (1) | 5 (5.1) | 2 (4.1) | 3 (6.1) |  |  |
| No (0) | 87 (88.8) | 42 (85.7) | 45 (91.8) |  |  |
| Unknown (2) | 6 (6.1) | 5 (10.2) | 1 (2.0) |  |  |
|  |  |  |  |  |  |
| **Wheezing, n (%)** |  |  |  | FE: p = 0.39 |  |
| Yes (1) | 6 (6.1) | 2 (4.1) | 4 (8.2) |  |  |
| No (0) | 87 (88.8) | 43 (87.8) | 44 (89.8) |  |  |
| Unknown (2) | 5 (5.1) | 4 (8.2) | 1 (2.0) |  |  |
|  |  |  |  |  |  |
| **Shortness of Breath, n (%)** |  |  |  | FE: p = 1.0 |  |
| Yes (1) | 79 (80.6) | 39 (79.6) | 40 (81.6) |  |  |
| No (0) | 18 (18.4) | 9 (18.4) | 9 (18.4) |  |  |
| Unknown (2) | 1 (1.0) | 1 (2.0) | 0 (0.0) |  |  |
|  |  |  |  |  |  |
| **Chest Pain, n (%)** |  |  |  | FE: p = 0.59 |  |
| Yes (1) | 29 (29.6) | 14 (28.6) | 15 (30.6) |  |  |
| No (0) | 67 (68.4) | 33 (67.3) | 34 (69.4) |  |  |
| Unknown (2) | 2 (2.0) | 2 (4.1) | 0 (0.0) |  |  |
|  |  |  |  |  |  |
| **Headache, n (%)** |  |  |  | FE: p = 0.01 |  |
| Yes (1) | 30 (30.6) | 19 (38.8) | 11 (22.4) |  |  |
| No (0) | 64 (65.3) | 26 (53.1) | 38 (77.6) |  |  |
| Unknown (2) | 4 (4.1) | 4 (8.2) | 0 (0.0) |  |  |
|  |  |  |  |  |  |
| **Loss of smell (Anosmia), n (%)** |  |  |  | FE: p = 0.53 |  |
| Yes (1) | 10 (10.2) | 4 (8.2) | 6 (12.2) |  |  |
| No (0) | 81 (82.6) | 40 (81.6) | 41 (83.7) |  |  |
| Unknown (2) | 7 (8.2) | 5 (10.2) | 2 (4.1) |  |  |
|  |  |  |  |  |  |
| **Loss of taste (Ageusia), n (%)** |  |  |  | FE: p = 0.64 |  |
| Yes (1) | 12 (12.2) | 6 (12.2) | 6 (12.2) |  |  |
| No (0) | 79 (80.6) | 38 (77.6) | 41 (83.7) |  |  |
| Unknown (2) | 7 (8.2) | 5 (10.2) | 2 (4.1) |  |  |
|  |  |  |  |  |  |
| **Seizures, n (%)** |  |  |  | FE: p = 0.12 |  |
| Yes (1) | 1 (1.0) | 1 (2.0) | 0 (0.0) |  |  |
| No (0) | 94 (95.9) | 45 (91.8) | 49 (100.0) |  |  |
| Unknown (2) | 3 (3.1) | 3 (6.1) | 0 (0.0) |  |  |
|  |  |  |  |  |  |
| **Fatigue/Malaise, n (%)** |  |  |  | FE: p = 0.39 |  |
| Yes (1) | 63 (64.3) | 28 (57.1) | 35 (71.4) |  |  |
| No (0) | 32 (32.6) | 19 (38.8) | 13 (26.5) |  |  |
| Unknown (2) | 3 (3.1) | 2 (4.1) | 1 (2.0) |  |  |
|  |  |  |  |  |  |
| **Anorexia, n (%)** |  |  |  | FE: p = 0.87 |  |
| Yes (1) | 13 (13.3) | 7 (14.3) | 6 (12.2) |  |  |
| No (0) | 78 (79.6) | 38 (77.5) | 40 (81.6) |  |  |
| Unknown (2) | 7 (8.2) | 4 (8.2) | 3 (6.1) |  |  |
|  |  |  |  |  |  |
| **Altered consciousness/confusion, n (%)** |  |  |  | FE: p = 0.12 |  |
| Yes (1) | 6 (6.1) | 4 (8.2) | 2 (4.1) |  |  |
| No (0) | 89 (90.8) | 42 (85.7) | 47 (95.9) |  |  |
| Unknown (2) | 3 (3.1) | 3 (6.1) | 0 (0.0) |  |  |
|  |  |  |  |  |  |
| **Muscle aches (myalgia), n (%)** |  |  |  | FE: p = 0.36 |  |
| Yes (1) | 49 (50.0) | 24 (49.0) | 25 (51.0) |  |  |
| No (0) | 46 (46.9) | 22 (44.9) | 24 (49.0) |  |  |
| Unknown (2) | 3 (3.1) | 3 (6.1) | 0 (0.0) |  |  |
|  |  |  |  |  |  |
| **Joint pain (arthralgia), n (%)** |  |  |  | FE: p = 0.04 |  |
| Yes (1) | 14 (14.3) | 5 (10.2) | 9 (18.4) |  |  |
| No (0) | 79 (80.6) | 39 (79.6) | 40 (81.6) |  |  |
| Unknown (2) | 5 (5.1) | 5 (10.2) | 0 (0.0) |  |  |
|  |  |  |  |  |  |
| **Abdominal pain, n (%)** |  |  |  | FE: p = 0.17 |  |
| Yes (1) | 20 (20.4) | 7 (14.3) | 13 (26.5) |  |  |
| No (0) | 73 (74.5) | 38 (77.5) | 35 (71.4) |  |  |
| Unknown (2) | 5 (5.1) | 4 (8.2) | 1 (2.0) |  |  |
|  |  |  |  |  |  |
| **Diarrhea, n (%)** |  |  |  | FE: p = 0.40 |  |
| Yes (1) | 34 (34.7) | 18 (36.7) | 16 (32.7) |  |  |
| No (0) | 62 (63.3) | 29 (59.2) | 33 (67.3) |  |  |
| Unknown (2) | 2 (2.0) | 2 (4.1) | 0 (0.0) |  |  |
|  |  |  |  |  |  |
| **Vomiting/Nausea, n (%)** |  |  |  | FE: p = 0.55 |  |
| Yes (1) | 30 (30.6) | 14 (28.6) | 16 (32.7) |  |  |
| No (0) | 66 (67.3) | 33 (67.3) | 33 (67.3) |  |  |
| Unknown (2) | 2 (2.0) | 2 (4.1) | 0 (0.0) |  |  |
|  |  |  |  |  |  |
| **Other symptom(s), n (%)** |  |  |  | FE: p = 1.0 |  |
| Yes (1) | 11 (11.2) | 5 (10.2) | 6 (12.2) |  |  |
| No (0) | 77 (78.6) | 39 (79.6) | 38 (77.5) |  |  |
| Unknown (2) | 10 (10.2) | 5 (10.2) | 5 (10.2) |  |  |

Supplementary Table S2. Patient signs and symptoms prior to hospital admission per treatment group.

Abbreviations: CS, Pearson's Chi-Square; FE, Fisher's Exact Test; WRS = Wilcoxon Rank Sum Test

| **PRE-ADMISSION MEDICATIONS** | | | | | |
| --- | --- | --- | --- | --- | --- |
|  | **Total Study Sample (n = 98)** | **Aggrenox (n = 49)** | **Standard of Care (n = 49)** |  |  |
| **ACE inhibitors, n (%)** |  |  |  | CS: p = 0.37 |  |
| Yes (1) | 13 (13.3) | 4 (8.2) | 9 (18.4) |  |  |
| No (0) | 68 (69.4) | 36 (73.5) | 32 (65.3) |  |  |
| Unknown (2) | 17 (17.4) | 9 (18.4) | 8 (16.3) |  |  |
|  |  |  |  |  |  |
| **ARBs, n (%)** |  |  |  | FE: p = 1.0 |  |
| Yes (1) | 3 (3.1) | 1 (2.0) | 2 (4.1) |  |  |
| No (0) | 78 (79.6) | 39 (79.6) | 39 (79.6) |  |  |
| Unknown (2) | 17 (17.3) | 9 (18.4) | 8 (16.3) |  |  |
|  |  |  |  |  |  |
| **NSAIDs, n (%)** |  |  |  | CS: p = 0.97 |  |
| Yes (1) | 29 (29.6) | 15 (30.6) | 14 (28.6) |  |  |
| No (0) | 53 (54.1) | 26 (53.1) | 27 (55.1) |  |  |
| Unknown (2) | 16 (16.3) | 8 (16.3) | 8 (16.3) |  |  |
|  |  |  |  |  |  |
| **Oral steroids, n (%)** |  |  |  | FE: p = 0.60 |  |
| Yes (1) | 7 (7.1) | 5 (10.2) | 2 (4.1) |  |  |
| No (0) | 75 (76.5) | 36 (73.5) | 39 (79.6) |  |  |
| Unknown (2) | 16 (16.3) | 8 (16.3) | 8 (16.3) |  |  |
|  |  |  |  |  |  |
| **Antivirals, n (%)** |  |  |  | FE: p = 1.0 |  |
| Yes (1) | 3 (3.1) | 2 (4.1) | 1 (2.0) |  |  |
| No (0) | 79 (80.6) | 39 (79.6) | 40 (81.6) |  |  |
| Unknown (2) | 16 (16.3) | 8 (16.3) | 8 (16.3) |  |  |
|  |  |  |  |  |  |
| **Antibiotics, n (%)** |  |  |  | FE: p = 0.89 |  |
| Yes (1) | 9 (9.2) | 5 (10.2) | 4 (8.2) |  |  |
| No (0) | 73 (74.5) | 37 (75.5) | 36 (73.5) |  |  |
| Unknown (2) | 16 (16.3) | 7 (14.3) | 9 (18.4) |  |  |
|  |  |  |  |  |  |
| **Other targeted COVID-19 Medications, n (%)** |  |  |  | FE: p = 1.0 |  |
| Yes (1) | 0 (0.0) | 0 (0.0) | 0 (0.0) |  |  |
| No (0) | 80 (81.6) | 40 (81.6) | 40 (81.6) |  |  |
| Unknown (2) | 18 (18.4) | 9 (18.4) | 9 (18.4) |  |  |

Supplementary Table S3. Pre-admission medication list per treatment group.

Abbreviations: CS, Pearson's Chi-Square; FE, Fisher's Exact Test

| **COVID Severity Linear Regression Models** | | | | | | | | |
| --- | --- | --- | --- | --- | --- | --- | --- | --- |
| Outcome Timeframe | Day 1 - Day 14  N = 64/98 | | | | Day 1 - Day 28  N = 63/96 | | | |
| Predictor Variables | β coef. | SE | P Value | β coef. | | | SE | P Value |
| Intercept | 3.841 | 1.867 | 0.045 | 4.119 | | | 1.873 | 0.033 |
| **Treatment (Aggrenox)** | 0.566 | 0.464 | 0.228 | 0.858 | | | 0.466 | 0.072 |
| **Demographics** |  |  |  |  | | |  |  |
| *Age* | -0.014 | 0.018 | 0.436 | -0.026 | | | 0.018 | 0.149 |
| *Male Sex* | -0.114 | 0.479 | 0.813 | 0.010 | | | 0.471 | 0.983 |
| **Risk Factors at Admission** |  |  |  |  | | |  |  |
| *Obesity* | 0.331 | 0.450 | 0.466 | -0.061 | | | 0.446 | 0.892 |
| *CPD* | -0.714 | 0.926 | 0.445 | -1.142 | | | 0.914 | 0.218 |
| *Type II diabetes* | -0.816 | 0.579 | 0.165 | -0.953 | | | 0.577 | 0.106 |
| *Cardiovascular Disease* | -0.453 | 1.127 | 0.690 | -1.066 | | | 1.107 | 0.341 |
| *Hypertension* | -0.375 | 0.527 | 0.479 | -0.042 | | | 0.527 | 0.937 |
| *AIDS/HIV* | 0.968 | 1.311 | 0.464 | 0.585 | | | 1.289 | 0.652 |
| *Chronic Kidney Disease* | 0.591 | 2.144 | 0.784 | 0.728 | | | 2.117 | 0.733 |
| *Current Smoker* | -1.171 | 0.784 | 0.142 | -1.252 | | | 0.771 | 0.111 |
| *Former Smoker* | -0.391 | 0.588 | 0.509 | 0.015 | | | 0.581 | 0.980 |
| *Diastolic Blood Pressure* | -0.014 | 0.021 | 0.489 | -0.011 | | | 0.021 | 0.586 |
| **Medications at Admission** |  |  |  |  | | |  |  |
| *NSAIDS* | 0.301 | 0.486 | 0.539 | 0.786 | | | 0.487 | 0.113 |
| *Oral Steroids* | 0.905 | 0.720 | 0.215 | 0.776 | | | 0.709 | 0.279 |
|  | R^2^ Value: 0.31 | | | | | R^2^ Value: 0.37 | | |

Supplementary Table S4.. All predictor variables related to risk factors or medications have a reference group of not having the event (no) compared to having the risk factor or taking the medication (yes).

| COMPLICATIONS (at ANY time during hospitalization) | | | | |
| --- | --- | --- | --- | --- |
|  | **Total Study Sample (n = 98)** | **Aggrenox (n = 49)** | **Standard of Care (n = 49)** |  |
| **Viral pneumonia/pneumonitis, n (%)** |  |  |  | FE: p = 1.0 |
| Yes (1) | 83 (84.7) | 42 (85.7) | 41 (83.7) |  |
| No (0) | 3 (3.1) | 1 (2.0) | 2 (4.1) |  |
| Unknown (2) | 12 (12.2) | 6 (12.2) | 6 (12.2) |  |
|  |  |  |  |  |
| **Bacterial pneumonia, n (%)** |  |  |  | FE: p = 0.44 |
| Yes (1) | 2 (2.0) | 0 (0.0) | 2 (4.1) |  |
| No (0) | 84 (85.7) | 42 (85.7) | 42 (85.7) |  |
| Unknown (2) | 12 (12.2) | 7 (14.3) | 5 (10.2) |  |
|  |  |  |  |  |
| **Acute Respiratory Distress Syndrome (ARDS), n (%)** |  |  |  | FE: p = 0.46 |
| Yes (1) | 9 (9.2) | 4 (8.2) | 5 (10.2) |  |
| No (0) | 84 (85.7) | 41 (83.7) | 43 (87.8) |  |
| Unknown (2) | 5 (5.1) | 4 (8.2) | 1 (2.0) |  |
|  |  |  |  |  |
| **Severity of ARDS, n (%)** | n = 9 | n = 4 | n = 5 | FE: p = 1.0 |
| Mild (1) | 0 (0.0) | 0 (0.0) | 0 (0.0) |  |
| Moderate (2) | 1 (11.1) | 0 (0.0) | 1 (20.0) |  |
| Severe (3) | 8 (88.9) | 4 (100.0) | 4 (80.0) |  |
| Unknown (4) | 0 (0.0) | 0 (0.0) | 0 (0.0) |  |
|  |  |  |  |  |
| **Pneumothorax, n (%)** |  |  |  | FE: p = 0.62 |
| Yes (1) | 1 (1.0) | 0 (0.0) | 1 (2.0) |  |
| No (0) | 93 (94.9) | 46 (93.9) | 47 (95.9) |  |
| Unknown (2) | 4 (4.1) | 3 (6.1) | 1 (2.0) |  |
|  |  |  |  |  |
| **Pleural effusion, n (%)** |  |  |  | FE: p = 0.62 |
| Yes (1) | 0 (0.0) | 0 (0.0) | 0 (0.0) |  |
| No (0) | 94 (95.9) | 46 (93.9) | 48 (98.0) |  |
| Unknown (2) | 4 (4.1) | 3 (6.1) | 1 (2.0) |  |
|  |  |  |  |  |
| **Cryptogenic organizing pneumonia (COP), n (%)** |  |  |  | FE: p = 0.62 |
| Yes (1) | 0 (0.0) | 0 (0.0) | 0 (0.0) |  |
| No (0) | 94 (95.9) | 46 (93.9) | 48 (98.0) |  |
| Unknown (2) | 4 (4.1) | 3 (6.1) | 1 (2.0) |  |
|  |  |  |  |  |
| **Bronchiolitis, n (%)** |  |  |  | FE: p = 0.36 |
| Yes (1) | 1 (1.0) | 1 (2.0) | 0 (0.0) |  |
| No (0) | 93 (94.9) | 45 (91.8) | 48 (98.0) |  |
| Unknown (2) | 4 (4.1) | 3 (6.1) | 1 (2.0) |  |
|  |  |  |  |  |
| **Cardiac arrest, n (%)** |  |  |  | FE: p = 0.52 |
| Yes (1) | 3 (3.1) | 2 (4.1) | 1 (2.0) |  |
| No (0) | 91 (92.9) | 44 (89.8) | 47 (95.9) |  |
| Unknown (2) | 4 (4.1) | 3 (6.1) | 1 (2.0) |  |
|  |  |  |  |  |
| **Myocardial infarction, n (%)** |  |  |  | FE: p = 0.24 |
| Yes (1) | 3 (3.1) | 0 (0.0) | 3 (6.1) |  |
| No (0) | 91 (92.9) | 46 (93.9) | 45 (91.8) |  |
| Unknown (2) | 4 (4.1) | 3 (6.1) | 1 (2.0) |  |
|  |  |  |  |  |
| **Cardiac ischemia, n (%)** |  |  |  | FE: p = 0.32 |
| Yes (1) | 5 (5.1) | 1 (2.0) | 4 (8.2) |  |
| No (0) | 89 (90.8) | 45 (91.8) | 44 (89.8) |  |
| Unknown (2) | 4 (4.1) | 3 (6.1) | 1 (2.0) |  |
|  |  |  |  |  |
| **Cardiac arrhythmia, n (%)** |  |  |  | FE: p = 0.52 |
| Yes (1) | 2 (2.0) | 1 (2.0) | 1 (2.0) |  |
| No (0) | 91 (92.9) | 44 (89.8) | 47 (95.9) |  |
| Unknown (2) | 5 (5.1) | 4 (8.2) | 1 (2.0) |  |
|  |  |  |  |  |
| **Myocarditis/Pericarditis, n (%)** |  |  |  | FE: p = 0.36 |
| Yes (1) | 0 (0.0) | 0 (0.0) | 0 (0.0) |  |
| No (0) | 93 (94.9) | 45 (91.8) | 48 (98.0) |  |
| Unknown (2) | 5 (5.1) | 4 (8.2) | 1 (2.0) |  |
|  |  |  |  |  |
| **Endocarditis, n (%)** |  |  |  | FE: p = 0.36 |
| Yes (1) | 0 (0.0) | 0 (0.0) | 0 (0.0) |  |
| No (0) | 93 (94.9) | 45 (91.8) | 48 (98.0) |  |
| Unknown (2) | 5 (5.1) | 4 (8.2) | 1 (2.0) |  |
|  |  |  |  |  |
| **Cardiomyopathy, n (%)** |  |  |  | FE: p = 0.36 |
| Yes (1) | 0 (0.0) | 0 (0.0) | 0 (0.0) |  |
| No (0) | 93 (94.9) | 45 (91.8) | 48 (98.0) |  |
| Unknown (2) | 5 (5.1) | 4 (8.2) | 1 (2.0) |  |
|  |  |  |  |  |
| **Congestive heart failure (CHF), n (%)** |  |  |  | FE: p = 0.52 |
| Yes (1) | 2 (2.0) | 1 (2.0) | 1 (2.0) |  |
| No (0) | 91 (92.9) | 44 (89.8) | 47 (95.9) |  |
| Unknown (2) | 5 (5.1) | 4 (8.2) | 1 (2.0) |  |
|  |  |  |  |  |
| **Seizure, n (%)** |  |  |  | FE: p = 0.62 |
| Yes (1) | 1 (1.0) | 0 (0.0) | 1 (2.0) |  |
| No (0) | 93 (94.9) | 46 (93.9) | 47 (95.9) |  |
| Unknown (2) | 4 (4.1) | 3 (6.1) | 1 (2.0) |  |
|  |  |  |  |  |
| **Stroke/Cerebrovascular accident, n (%)** |  |  |  | FE: p = 0.62 |
| Yes (1) | 0 (0.0) | 0 (0.0) | 0 (0.0) |  |
| No (0) | 94 (95.9) | 46 (93.9) | 48 (98.0) |  |
| Unknown (2) | 4 (4.1) | 3 (6.1) | 1 (2.0) |  |
|  |  |  |  |  |
| **Meningitis/Encephalitis, n (%)** |  |  |  | FE: p = 0.62 |
| Yes (1) | 0 (0.0) | 0 (0.0) | 0 (0.0) |  |
| No (0) | 94 (95.9) | 46 (93.9) | 48 (98.0) |  |
| Unknown (2) | 4 (4.1) | 3 (6.1) | 1 (2.0) |  |
|  |  |  |  |  |
| **Bacteremia, n (%)** |  |  |  | FE: p = 0.78 |
| Yes (1) | 7 (7.1) | 4 (8.2) | 3 (6.1) |  |
| No (0) | 88 (89.8) | 43 (87.8) | 45 (91.8) |  |
| Unknown (2) | 3 (3.1) | 2 (4.1) | 1 (2.0) |  |
|  |  |  |  |  |
| **Coagulation disorder/DIC, n (%)** |  |  |  | FE: p = 0.24 |
| Yes (1) | 3 (3.1) | 0 (0.0) | 3 (6.1) |  |
| No (0) | 91 (92.9) | 46 (93.9) | 45 (91.8) |  |
| Unknown (2) | 4 (4.1) | 3 (6.1) | 1 (2.0) |  |
|  |  |  |  |  |
| **Pulmonary embolism (PE), n (%)** |  |  |  | FE: p = 1.0 |
| Yes (1) | 1 (1.0) | 0 (0.0) | 1 (2.0) |  |
| No (0) | 94 (95.9) | 47 (95.9) | 47 (95.9) |  |
| Unknown (2) | 3 (3.1) | 2 (4.1) | 1 (2.0) |  |
|  |  |  |  |  |
| **Anemia, n (%)** |  |  |  | FE: p = 0.59 |
| Yes (1) | 13 (13.3) | 7 (14.3) | 6 (12.2) |  |
| No (0) | 81 (82.6) | 39 (79.6) | 42 (85.7) |  |
| Unknown (2) | 4 (4.1) | 3 (6.1) | 1 (2.0) |  |
|  |  |  |  |  |
| **Rhabdomyolysis/Myositis, n (%)** |  |  |  | FE: p = 1.0 |
| Yes (1) | 0 (0.0) | 0 (0.0) | 0 (0.0) |  |
| No (0) | 95 (96.9) | 47 (95.9) | 48 (98.0) |  |
| Unknown (2) | 3 (3.1) | 2 (4.1) | 1 (2.0) |  |
|  |  |  |  |  |
| **Acute renal injury/Acute renal failure, n (%)** |  |  |  | FE: p = 0.54 |
| Yes (1) | 14 (14.3) | 5 (10.2) | 9 (18.4) |  |
| No (0) | 81 (82.6) | 42 (85.7) | 39 (79.6) |  |
| Unknown (2) | 3 (3.1) | 2 (4.1) | 1 (2.0) |  |
|  |  |  |  |  |
| **Gastrointestinal hemorrhage, n (%)** |  |  |  | FE: p = 1.0 |
| Yes (1) | 1 (1.0) | 0 (0.0) | 1 (2.0) |  |
| No (0) | 94 (95.9) | 47 (95.9) | 47 (95.9) |  |
| Unknown (2) | 3 (3.1) | 2 (4.1) | 1 (2.0) |  |
|  |  |  |  |  |
| **Pancreatitis, n (%)** |  |  |  | FE: p = 1.0 |
| Yes (1) | 2 (2.0) | 1 (2.0) | 1 (2.0) |  |
| No (0) | 93 (94.9) | 46 (93.9) | 47 (95.9) |  |
| Unknown (2) | 3 (3.1) | 2 (4.1) | 1 (2.0) |  |
|  |  |  |  |  |
| **Liver dysfunction, n (%)** |  |  |  | FE: p = 0.71 |
| Yes (1) | 21 (21.4) | 9 (18.4) | 12 (14.5) |  |
| No (0) | 74 (75.5) | 38 (77.5) | 36 (73.5) |  |
| Unknown (2) | 3 (3.1) | 2 (4.1) | 1 (2.0) |  |
|  |  |  |  |  |
| **Hyperglycemia, n (%)** |  |  |  | FE: p = 0.40 |
| Yes (1) | 21 (21.4) | 12 (24.5) | 9 (18.4) |  |
| No (0) | 73 (74.5) | 34 (69.4) | 39 (79.6) |  |
| Unknown (2) | 4 (4.1) | 3 (6.1) | 1 (2.0) |  |
|  |  |  |  |  |
| **Hypoglycemia, n (%)** |  |  |  | FE: p = 0.32 |
| Yes (1) | 5 (5.1) | 1 (2.0) | 4 (8.2) |  |
| No (0) | 89 (90.8) | 45 (91.8) | 44 (89.8) |  |
| Unknown (2) | 4 (4.1) | 3 (6.1) | 1 (2.0) |  |
|  |  |  |  |  |
| **Other complication(s), n (%)** |  |  |  | FE: p = 0.60 |
| Yes (1) | 20 (20.4) | 8 (16.3) | 12 (24.5) |  |
| No (0) | 70 (71.4) | 37 (75.5) | 33 (67.3) |  |
| Unknown (2) | 8 (8.2) | 4 (8.2) | 4 (8.2) |  |

Table S5. List of complications per treatment group.

Abbreviations: CS, Pearson's Chi-Square; DIC, Disseminated Intravascular Coagulation; FE, Fisher's Exact Test

|  | **Total Study Sample (n = 98)** | **Group 1:  Alive at Day 28 (n = 90)** | **Group 2:  Dead at Day 28 (n = 8)** |  |  |
| --- | --- | --- | --- | --- | --- |
| SECONDARY OUTCOMES: LAB VALUES | | | | | |
|  | Median [Q1, Q3] | | |  |  |
| **Hemoglobin @ Day 1** | 13.2 [12, 14.5] | 13.3 [12.1, 14.5] | 11.4 [11.0, 15.8] | WRS: p = 0.52 |  |
|  |  |  |  |  |  |
| **Change in Hemoglobin: Day 1 -> Day 7 (Day 1 - Day 7)** | 0.1 [-0.2, 1.0] | 0.1 [-0.2, 0.9] | 1.6 [-0.7, 3.8] | WRS: p = 0.78 |  |
|  |  |  |  |  |  |
| **WBC count @ Day 1** | 6.8 [4.8, 10.0] | 6.8 [4.7, 9.6] | 8.4 [6.0, 12.3] | WRS: p = 0.28 |  |
|  |  |  |  |  |  |
| **Change in WBC count: Day 1 -> Day 7 (Day 1 - Day 7)** | -2.8 [-4.3, 1.3] | -2.8 [-4.2, 1.8] | -2.8 [-6.7, 1.2] | WRS: p = 0.78 |  |
|  |  |  |  |  |  |
| **Lymphocyte count @ Day 1** | 1.0 [0.6, 1.7] | 1.0 [0.7, 1.7] | 0.5 [0.4, 0.7] | WRS: p = 0.002 |  |
|  |  |  |  |  |  |
| **Change in Lymphocyte count: Day 1 -> Day 7 (Day 1 - Day 7)** | -0.4 [-1.0, 0.4] | -0.4 [-2.2, 0.3] | 0.0 [-0.4, 0.4] | WRS: p = 0.69 |  |
|  |  |  |  |  |  |
| **Neutrophil count @ Day 1** | 5.7 [3.6, 10.1] | 5.6 [3.3, 9.6] | 7.6 [4.7, 11.3] | WRS: p = 0.44 |  |
|  |  |  |  |  |  |
| **Change in Neutrophil count: Day 1 -> Day 7 (Day 1 - Day 7)** | -4.1 [-6.3, 1.5] | -4.1 [-8.0, 2.7] | -2.8 [-6.3, 0.7] | WRS: p = 0.79 |  |
|  |  |  |  |  |  |
| **Hematocrit @ Day 1** | 39.7 [36.2, 43.1] | 39.8 [37.0, 43.1] | 34.7 [33.6, 46.8] | WRS: p = 0.64 |  |
|  |  |  |  |  |  |
| **Change in Hematocrit: Day 1 -> Day 7 (Day 1 - Day 7)** | 0.9 [0.1, 3.4] | 0.9 [0.1, 3.3] | 4.6 [-3.5, 12.7] | WRS: p = 0.87 |  |
|  |  |  |  |  |  |
| **Platelets @ Day 1** | 216.0 [170.0, 276.0] | 234.0 [182.0, 276.0] | 161.0 [150.0, 175.0] | WRS: p = 0.03 |  |
|  |  |  |  |  |  |
| **Change in Platelets: Day 1 -> Day 7 (Day 1 - Day 7)** | -71.0 [-140.0, -34.0] | -99.0 [-158.0, -37.0] | 88.5 [33.0, 144.0] | WRS: p = 0.03 |  |
|  |  |  |  |  |  |
| **APTT @ Day 1** | 32.0 [29.2, 34.0] | 32.0 [29.9, 33.7] | 28.8 [26.4, 35.7] | WRS: p = 0.63 |  |
|  |  |  |  |  |  |
| **Change in APTT: Day 1 -> Day 7 (Day 1 - Day 7)** | 1.2 [1.2, 1.2] | 1.2 [1.2, 1.2] | NA | NA |  |
|  |  |  |  |  |  |
| **PT @ Day 1** | 13.2 [12.8, 13.8] | 13.1 [12.8, 13.4] | 14.6 [13.0, 15.3] | WRS: p = 0.13 |  |
|  |  |  |  |  |  |
| **Change in PT: Day 1 -> Day 7 (Day 1 - Day 7)** | -1.1 [-1.1, -1.1] | -1.1 [-1.1, -1.1] | NA | NA |  |
|  |  |  |  |  |  |
| **INR @ Day 1** | 1.0 [1.0, 1.0] | 1.0 [1.0, 1.0] | 1.2 [1.0, 1.2] | WRS: p = 0.03 |  |
|  |  |  |  |  |  |
| **Change in INR: Day 1 -> Day 7 (Day 1 - Day 7)** | -0.2 [-0.2, -0.2] | -0.2 [-0.2, -0.2] | NA | NA |  |
|  |  |  |  |  |  |
| **ALT/SGPT @ Day 1** | 35.0 [22.0, 53.0] | 35.0 [22.0, 53.5] | 32.0 [18.0, 38.0] | WRS: p = 0.60 |  |
|  |  |  |  |  |  |
| **Change in ALT/SGPT: Day 1 -> Day 7 (Day 1 - Day 7)** | -7.0 [-28.0, 10.0] | -7.0 [-28.0, 9.0] | 10.0 [10.0, 10.0] | WRS: p = 0.39 |  |
|  |  |  |  |  |  |
| **Total Bilirubin @ Day 1** | 0.5 [0.3, 0.6] | 0.5 [0.3, 0.6] | 0.5 [0.5, 0.5] | WRS: p = 0.85 |  |
|  |  |  |  |  |  |
| **Change in Total Bilirubin: Day 1 -> Day 7 (Day 1 - Day 7)** | 0.2 [-0.1, 0.3] | 0.2 [0.0, 0.3] | -0.1 [-0.1, -0.1] | WRS: p = 0.32 |  |
|  |  |  |  |  |  |
| **AST/SGOT @ Day 1** | 44.0 [26.0, 62.0] | 44.0 [27.0, 61.5] | 26.0 [26.0, 71.0] | WRS: p = 0.81 |  |
|  |  |  |  |  |  |
| **Change in AST/SGOT: Day 1 -> Day 7 (Day 1 - Day 7)** | 17.5 [-11.0, 29.0] | 19.0 [0.0, 29.0] | -19.0 [-19.0, -19.0] | WRS: p = 0.17 |  |
|  |  |  |  |  |  |
| **Glucose @ Day 1** | 122.0 [103.5, 159.0] | 118.0 [102.5, 141.5] | 360.0 [239.5, 429.0] | WRS: p < 0.001 |  |
|  |  |  |  |  |  |
| **Change in Glucose: Day 1 -> Day 7 (Day 1 - Day 7)** | 23.0 [1.5, 36.0] | 20.5 [-5.0, 31.0] | 189.0 [167.0, 211.0] | WRS: p = 0.02 |  |
| **Urea [BUN] @ Day 1** | 14.0 [11.0, 18.5] | 13.0 [11.0, 17.0] | 32.5 [21.5, 55.5] | WRS: p < 0.001 |  |
|  |  |  |  |  |  |
| **Change in Urea [BUN]: Day 1 -> Day 7 (Day 1 - Day 7)** | -4.0 [-9.0, 10.5] | -5.5 [-9.0, -1.0] | 30.5 [11.0, 50.0] | WRS: p = 0.051 |  |
|  |  |  |  |  |  |
| **Lactate @ Day 1** | 1.8 [1.1, 2.4] | 1.7 [0.9, 2.2] | 1.8 [1.5, 2.6] | WRS: p = 0.47 |  |
|  |  |  |  |  |  |
| **Change in Lactate: Day 1 -> Day 7 (Day 1 - Day 7)** | 0.4 [0.4, 0.4] | 0.4 [0.4, 0.4] | NA | NA |  |
|  |  |  |  |  |  |
| **Creatinine @ Day 1** | 0.8 [0.7, 1.1] | 0.8 [0.7, 1.0] | 1.8 [1.2, 2.4] | WRS: p = 0.001 |  |
|  |  |  |  |  |  |
| **Change in Creatinine: Day 1 -> Day 7 (Day 1 - Day 7)** | 0.1 [0.0, 0.3] | 0.1 [0.1, 0.3] | -4.7 [-7.7, -1.6] | WRS: p = 0.02 |  |
|  |  |  |  |  |  |
| **Sodium @ Day 1** | 136.0 [133.0, 139.0] | 136.0 [134.0, 139.0] | 131.0 [130.0, 138.5] | WRS: p = 0.15 |  |
|  |  |  |  |  |  |
| **Change in Sodium: Day 1 -> Day 7 (Day 1 - Day 7)** | -2.0 [-5.5, 2.0] | -1.5 [-3.0, 4.0] | -14.5 [-17.0, -12.0] | WRS: p = 0.03 |  |
|  |  |  |  |  |  |
| **Potassium @ Day 1** | 4.1 [3.8, 4.5] | 4.1 [3.8, 4.4] | 4.4 [4.2, 4.9] | WRS: p = 0.04 |  |
|  |  |  |  |  |  |
| **Change in Potassium: Day 1 -> Day 7 (Day 1 - Day 7)** | -0.3 [-0.7, -0.2] | -0.3 [-0.5, -0.1] | -1.2 [-1.6, -0.7] | WRS: p = 0.06 |  |
|  |  |  |  |  |  |
| **Procalcitonin @ Day 1** | 0.1 [0.1, 0.3] | 0.1 [0.1, 0.3] | 1.1 [1.1, 1.1] | WRS: p = 0.11 |  |
|  |  |  |  |  |  |
| **Change in Procalcitonin: Day 1 -> Day 7 (Day 1 - Day 7)** | 1.8 [-0.3, 3.9] | 1.8 [-0.3, 3.9] | NA | NA |  |
|  |  |  |  |  |  |
| **CRP @ Day 1** | 82.0 [48.0, 121.0] | 78.5 [46.0, 111.0] | 186.0 [134.5, 194.5] | WRS: p = 0.03 |  |
|  |  |  |  |  |  |
| **Change in CRP: Day 1 -> Day 7 (Day 1 - Day 7)** | 101.0 [85.0, 165.0] | 101.0 [85.0, 165.0] | NA | NA |  |
|  |  |  |  |  |  |
| **LDH @ Day 1** | 420.0 [293.0, 549.0] | 400.5 [287.0, 521.0] | 716.0 [663.0, 959.0] | WRS: p = 0.01 |  |
|  |  |  |  |  |  |
| **Change in LDH: Day 1 -> Day 7 (Day 1 - Day 7)** | 186.0 [71.5, 308.0] | 168.0 [52.0, 275.0] | 402.0 [402.0, 402.0] | WRS: p = 0.13 |  |
|  |  |  |  |  |  |
| **Kinase @ Day 1** | 120.0 [58.0, 201.5] | 120.0 [53.0, 150.0] | 337.5 [72.0, 603.0] | WRS: p = 0.39 |  |
|  |  |  |  |  |  |
| **Change in Kinase: Day 1 -> Day 7 (Day 1 - Day 7)** | NA | NA | NA | NA |  |
|  |  |  |  |  |  |
| **Troponin @ Day 1** | 0.01 [0.01, 0.02] | 0 [0, 0] | 0.1 [0, 0.4] | WRS: p = 0.01 |  |
|  |  |  |  |  |  |
| **Change in Troponin: Day 1 -> Day 7 (Day 1 - Day 7)** | -0.09 [-0.09, -0.09] | -0.09 [-0.09, -0.09] | NA | NA |  |
|  |  |  |  |  |  |
| **D-Dimer @ Day 1** | 914.0 [626.0, 1231.0] | 878.0 [603.0, 1154.0] | 1680.0 [1233.0, 4104.0] | WRS: p = 0.01 |  |
|  |  |  |  |  |  |
| **Change in D-Dimer: Day 1 -> Day 7 (Day 1 - Day 7)** | 114.0 [-1408.0, 336.0] | 114.0 [-1069.5, 288.0] | -1676.0 [-4119.0, 767.0] | WRS: p = 0.79 |  |
|  |  |  |  |  |  |
| **Ferritin @ Day 1** | 572.0 [293.0, 1183.5] | 562.0 [286.0, 1180.0] | 983.0 [575.0, 1187.0] | WRS: p = 0.29 |  |
|  |  |  |  |  |  |
| **Change in Ferritin: Day 1 -> Day 7 (Day 1 - Day 7)** | 381.0 [279.0, 960.0] | 451.0 [279.0, 960.0] | 345.0 [345.0, 345.0] | WRS: p = 0.62 |  |

Supplementary Table S6. Differences in laboratory markers at hospital admission and through day 7 between *survivors and non-survivors*.

Abbreviations: Q1 = Quartile 1; Q3 = Quartile 3; WRS = Wilcoxon Rank Sum Test

|  | **Group 1:  No Respiratory Failure at Day 28 (n = 85)** | **Group 2:  Respiratory Failure at Day 28 (n = 11)** |  |
| --- | --- | --- | --- |
| SECONDARY OUTCOMES: LAB VALUES | | | |
|  | Median [Q1, Q3] | |  |
| **Hemoglobin @ Day 1** | 13.2 [12.1, 14.5] | 14.1 [11.0, 15.8] | WRS: p = 0.81 |
|  |  |  |  |
| **Change in Hemoglobin: Day 1 -> Day 7 (Day 1 - Day 7)** | 0.1 [-0.2, 0.7] | -0.4 [-1.0, 1.9] | WRS: p = 0.36 |
|  |  |  |  |
| **WBC count @ Day 1** | 6.8 [4.5, 9.2] | 11.0 [6.8, 13.3] | WRS: p = 0.02 |
|  |  |  |  |
| **Change in WBC count: Day 1 -> Day 7 (Day 1 - Day 7)** | -1.9 [-3.2, 2.2] | -5.4 [-7.9, -1.4] | WRS: p = 0.09 |
|  |  |  |  |
| **Lymphocyte count @ Day 1** | 1.0 [0.7, 1.7] | 0.7 [0.5, 0.7] | WRS: p = 0.004 |
|  |  |  |  |
| **Change in Lymphocyte count: Day 1 -> Day 7 (Day 1 - Day 7)** | -0.4 [-1.0, -0.2] | -0.4 [-3.4, 0.4] | WRS: p = 0.70 |
|  |  |  |  |
| **Neutrophil count @ Day 1** | 5.6 [3.2, 9.6] | 8.1 [5.2, 11.0] | WRS: p = 0.30 |
|  |  |  |  |
| **Change in Neutrophil count: Day 1 -> Day 7 (Day 1 - Day 7)** | -0.9 [-4.9, 3.9] | -6.3 [-81.1, 0.7] | WRS: p = 0.12 |
|  |  |  |  |
| **Hematocrit @ Day 1** | 39.8 [37.0, 43.1] | 41.1 [33.7, 46.8] | WRS: p = 0.74 |
|  |  |  |  |
| **Change in Hematocrit: Day 1 -> Day 7 (Day 1 - Day 7)** | 1.4 [0.3, 3.3] | -3.3 [-4.8, 4.9] | WRS: p = 0.16 |
|  |  |  |  |
| **Platelets @ Day 1** | 234.0 [182.0, 276.0] | 162.0 [150.0, 200.0] | WRS: p = 0.048 |
|  |  |  |  |
| **Change in Platelets: Day 1 -> Day 7 (Day 1 - Day 7)** | -108.0 [-166.0, -50.5] | -0.5 [-39.5, 88.5] | WRS: p = 0.02 |
|  |  |  |  |
| **APTT @ Day 1** | 32.1 [29.9, 33.7] | 30.4 [26.4, 35.7] | WRS: p = 0.67 |
|  |  |  |  |
| **Change in APTT: Day 1 -> Day 7 (Day 1 - Day 7)** | 1.2 [1.2, 1.2] | NA | NA |
|  |  |  |  |
| **PT @ Day 1** | 13.0 [12.8, 13.4] | 14.3 [13.0, 15.3] | WRS: p = 0.06 |
|  |  |  |  |
| **PT @ Day 7** | 15.2 [14.3, 16.1] | 19.2 [19.2, 19.2] | WRS: p = 0.22 |
|  |  |  |  |
| **Change in PT: Day 1 -> Day 7 (Day 1 - Day 7)** | -1.1 [-1.1, -1.1] | NA | NA |
|  |  |  |  |
| **INR @ Day 1** | 1.0 [1.0, 1.0] | 1.1 [1.0, 1.2] | WRS: p = 0.06 |
|  |  |  |  |
| **Change in INR: Day 1 -> Day 7 (Day 1 - Day 7)** | -0.2 [-0.2, -0.2] | NA | NA |
|  |  |  |  |
| **ALT/SGPT @ Day 1** | 35.0 [21.5, 54.0] | 37.0 [25.0, 49.0] | WRS: p = 0.90 |
|  |  |  |  |
| **Change in ALT/SGPT: Day 1 -> Day 7 (Day 1 - Day 7)** | -7.0 [-28.0, 9.0] | 10.0 [-14.0, 18.0] | WRS: p = 0.31 |
|  |  |  |  |
| **Total Bilirubin @ Day 1** | 0.5 [0.3, 0.6] | 0.5 [0.5, 0.6] | WRS: p = 0.37 |
|  |  |  |  |
| **Change in Total Bilirubin: Day 1 -> Day 7 (Day 1 - Day 7)** | 0.3 [0.1, 0.3] | -0.1 [-0.2, -0.1] | WRS: p = 0.01 |
|  |  |  |  |
| **AST/SGOT @ Day 1** | 43.0 [26.5, 59.0] | 58.5 [26.0, 73.0] | WRS: p = 0.40 |
|  |  |  |  |
| **Change in AST/SGOT: Day 1 -> Day 7 (Day 1 - Day 7)** | 20.0 [0.0, 39.0] | 16.0 [-19.0, 22.0] | WRS: p = 0.40 |
|  |  |  |  |
| **Glucose @ Day 1** | 118.0 [102.0, 140.0] | 331.0 [174.0, 431.0] | WRS: p < 0.001 |
|  |  |  |  |
| **Change in Glucose: Day 1 -> Day 7 (Day 1 - Day 7)** | 25.0 [-5.0, 33.0] | 93.5 [17.5, 189.0] | WRS: p = 0.23 |
|  |  |  |  |
| **Urea [BUN] @ Day 1** | 13.0 [11.0, 17.0] | 25.0 [18.0, 51.0] | WRS: p < 0.001 |
|  |  |  |  |
| **Change in Urea [BUN]: Day 1 -> Day 7 (Day 1 - Day 7)** | -4.0 [-9.0, 10.0] | 3.5 [-9.0, 30.5] | WRS: p = 0.45 |
|  |  |  |  |
| **Lactate @ Day 1** | 1.2 [0.9, 2.1] | 2.8 [1.7, 3.5] | WRS: p = 0.02 |
|  |  |  |  |
|  |  |  |  |
| **Change in Lactate: Day 1 -> Day 7 (Day 1 - Day 7)** | NA | 0.4 [0.4, 0.4] | NA |
|  |  |  |  |
|  |  |  |  |
| **Creatinine @ Day 1** | 0.8 [0.7, 1.0] | 1.2 [0.9, 2.4] | WRS: p = 0.01 |
|  |  |  |  |
| **Change in Creatinine: Day 1 -> Day 7 (Day 1 - Day 7)** | 0.1 [0.1, 0.3] | -0.9 [-4.7, 0.1] | WRS: p = 0.09 |
|  |  |  |  |
| **Sodium @ Day 1** | 136.0 [134.0, 139.0] | 132.0 [130.0, 137.0] | WRS: p = 0.10 |
|  |  |  |  |
| **Change in Sodium: Day 1 -> Day 7 (Day 1 - Day 7)** | -2.0 [-5.0, 4.0] | -7.5 [-14.5, -1.5] | WRS: p = 0.13 |
|  |  |  |  |
| **Potassium @ Day 1** | 4.1 [3.8, 4.4] | 4.3 [4.0, 4.8] | WRS: p = 0.12 |
|  |  |  |  |
| **Change in Potassium: Day 1 -> Day 7 (Day 1 - Day 7)** | -0.2 [-0.5, 0.1] | -1.0 [-1.4, -0.6] | WRS: p = 0.02 |
|  |  |  |  |
| **Procalcitonin @ Day 1** | 0.1 [0.1, 0.2] | 0.4 [0.2, 1.1] | WRS: p = 0.04 |
|  |  |  |  |
| **Change in Procalcitonin: Day 1 -> Day 7 (Day 1 - Day 7)** | 3.9 [3.9, 3.9] | -0.3 [-0.3, -0.3] | NA |
|  |  |  |  |
| **CRP @ Day 1** | 75.5 [42.0, 107.0] | 178.0 [103.0, 195.0] | WRS: p = 0.004 |
|  |  |  |  |
| **Change in CRP: Day 1 -> Day 7 (Day 1 - Day 7)** | 133.0 [92.0, 213.5] | 85.0 [85.0, 85.0] | WRS: p = 0.48 |
|  |  |  |  |
| **LDH @ Day 1** | 391.5 [284.0, 482.0] | 689.5 [603.0, 911.0] | WRS: p < 0.001 |
|  |  |  |  |
| **Change in LDH: Day 1 -> Day 7 (Day 1 - Day 7)** | 186.0 [91.0, 275.0] | 139.5 [-123.0, 402.0] | WRS: p = 1.0 |
|  |  |  |  |
| **Kinase @ Day 1** | 120.0 [53.0, 150.0] | 337.5 [72.0, 603.0] | WRS: p = 0.39 |
|  |  |  |  |
| **Change in Kinase: Day 1 -> Day 7 (Day 1 - Day 7)** | NA | NA | NA |
|  |  |  |  |
| **Troponin @ Day 1** | 0.01 [0.01, 0.01] | 0.03 [0.01, 0.3] | WRS: p = 0.004 |
|  |  |  |  |
| **Change in Troponin: Day 1 -> Day 7 (Day 1 - Day 7)** | NA | NA | NA |
|  |  |  |  |
| **D-Dimer @ Day 1** | 850.0 [586.5, 1148.0] | 1390.5 [1113.0, 2958.0] | WRS: p = 0.01 |
|  |  |  |  |
| **Change in D-Dimer: Day 1 -> Day 7 (Day 1 - Day 7)** |  |  | WRS: p = 0.44 |
|  |  |  |  |
| **Ferritin @ Day 1** | 553.0 [272.0, 1180.0] | 903.5 [647.5, 1315.0] | WRS: p = 0.14 |
|  |  |  |  |
| **Change in Ferritin: Day 1 -> Day 7 (Day 1 - Day 7)** | 381.0 [279.0, 960.0] | 433.0 [345.0, 521.0] | WRS: p = 1.0 |

Supplementary Table S7. Differences in laboratory markers at hospital admission and through day 7 between patients *with and without respiratory failure*.

Abbreviations: Q1 = Quartile 1; Q3 = Quartile 3; WRS = Wilcoxon Rank Sum Test

|  | **Total Study Sample (n = 98)** | **Aggrenox (n = 49)** | **Standard of Care (n = 49)** | P-Value |  |
| --- | --- | --- | --- | --- | --- |
| **Laboratory Markers** | | | | | |
|  | Median [Q1, Q3] | | |  |  |
| **Change in Hemoglobin: Day 1 -> Day 7** | 0.1 [-0.2, 1.0] | 0.6 [-0.2, 1.3] | 0.1 [-0.2, 0.2] | WRS: p = 0.48 |  |
|  |  |  |  |  |  |
| **Change in WBC count: Day 1 -> Day 7** | -2.8 [-4.3, 1.3] | -2.8 [-6.7, 1.3] | -2.6 [-4.2, 2.6] | WRS: p = 0.72 |  |
|  |  |  |  |  |  |
| **Change in Lymphocyte count: Day 1 -> Day 7** | -0.4 [-1.0, 0.4] | -0.4 [-0.4, -0.2] | -1.5 [-3.4, 0.7] | WRS: p = 0.83 |  |
|  |  |  |  |  |  |
| **Change in Neutrophil count: Day 1 -> Day 7** | -4.1 [-6.3, 1.5] | -4.1 [-6.3, 1.5] | -2.5 [-43.4, 3.7] | WRS: p = 1.0 |  |
|  |  |  |  |  |  |
| **Change in Hematocrit: Day 1 -> Day 7** | 0.9 [0.1, 3.4] | 2.7 [1.4, 3.4] | 0.1 [-3.0, 0.6] | WRS: p = 0.08 |  |
|  |  |  |  |  |  |
| **Change in Platelets: Day 1 -> Day 7** | -71.0 [-140.0, -34.0] | -101.0 [-158.0, -37.0] | -45.0 [-113.0, -12.0] | WRS: p = 0.33 |  |
|  |  |  |  |  |  |
| **Change in ALT/SGPT: Day 1 -> Day 7** | -7.0 [-28.0, 10.0] | -9.5 [-114.0, 12.0] | -7.0 [-16.5, 3.5] | WRS: p = 0.80 |  |
|  |  |  |  |  |  |
| **Change in Total Bilirubin: Day 1 -> Day 7** | 0.2 [-0.1, 0.3] | 0.2 [0.0, 0.4] | 0.2 [-0.1, 0.3] | WRS: p = 0.44 |  |
|  |  |  |  |  |  |
| **Change in AST/SGOT: Day 1 -> Day 7** | 17.5 [-11.0, 29.0] | 20.0 [-12.0, 29.0] | 11.5 [-5.5, 30.5] | WRS: p = 0.90 |  |
|  |  |  |  |  |  |
| **Change in Glucose: Day 1 -> Day 7** | 23.0 [1.5, 36.0] | 27.5 [-5.0, 39.0] | 17.5 [8.0, 33.0] | WRS: p = 0.60 |  |
|  |  |  |  |  |  |
| **Change in Urea [BUN]: Day 1 -> Day 7** | -4.0 [-9.0, 10.5] | -5.5 [-9.0, 10.0] | -4.0 [-9.0, 11.0] | WRS: p = 0.82 |  |
|  |  |  |  |  |  |
| **Change in Creatinine: Day 1 -> Day 7** | 0.1 [0.0, 0.3] | 0.1 [0.0, 0.2] | 0.1 [0.0, 0.3] | WRS: p = 0.97 |  |
|  |  |  |  |  |  |
| **Change in Sodium: Day 1 -> Day 7** | -2.0 [-5.5, 2.0] | -1.5 [-3.0, -1.0] | -2.5 [-7.0, 4.0] | WRS: p = 0.73 |  |
|  |  |  |  |  |  |
| **Change in Potassium: Day 1 -> Day 7** | -0.3 [-0.7, -0.2] | -0.3 [-0.5, -0.2] | -0.4 [-1.2, 0.1] | WRS: p = 0.79 |  |
|  |  |  |  |  |  |
| **Change in CRP: Day 1 -> Day 7** | 101.0 [85.0, 165.0] | 92.0 [83.0, 101.0] | 165.0 [85.0, 262.0] | WRS: p = 0.25 |  |
|  |  |  |  |  |  |
| **Change in LDH: Day 1 -> Day 7** | 186.0 [71.5, 308.0] | 204.0 [168.0, 402.0] | 91.0 [52.0, 275.0] | WRS: p = 0.30 |  |
|  |  |  |  |  |  |
| **Change in D-Dimer: Day 1 -> Day 7** | 114.0 [-1408.0, 336.0] | -197.5 [-2425.0, 366.0] | 114.0 [-1408.0, 240.0] | WRS: p = 0.83 |  |
|  |  |  |  |  |  |
| **Change in Ferritin: Day 1 -> Day 7** | 381.0 [279.0, 960.0] | 652.5 [312.0, 1752.0] | 381.0 [115.0, 521.0] | WRS: p = 0.48 |  |

Supplementary Table S8 Laboratory inflammatory markers per treatment group.

Abbreviations: ALT/SGPT, alanine transaminase/serum glutamic-pyruvic transaminase; AST/SGOT; aspartate aminotransferase/serum glutamic-oxaloacetic transaminase; BUN, blood urea nitrogen; CS, Pearson's Chi-Square; CRP, C-Reactive Protein; FE, Fisher's Exact Test; LDH, lactate dehydrogenase; Q1 = Quartile 1; Q3 = Quartile 3; TT = T-Test; WBC, White blood cells; WRS = Wilcoxon Rank Sum Test
