# Supplementary material for: A randomized controlled trial to evaluate outcomes with Aggrenox in patients with SARS-CoV-2 infection
Source: PLoS One. 2023 Jan 30;18(1):e0274243. doi: 10.1371/journal.pone.0274243 (PMC9886260; doi:10.1371/journal.pone.0274243)
Supplement: S2 File — (PDF) [file pone.0274243.s002.pdf]

INTERVENTIONAL
RESEARCH PROTOCOL TEMPLATE

(HRP-503a)

STUDY INFORMATION

- **Title of Project:**
  A randomized controlled trial to evaluate the outcomes with Aggrenox in patients with SARS-CoV-2 infection

- **Principal Investigators Name**
  Amit Singla, MD, FAANS

- **Principal Investigator Div. & Dept.**
  Neurosurgery

- **Principal Investigator Contact Info:**
  E mail: As3321@njms.rutgers.edu
  Address: DOC 8th Floor, 90 Bergen Street, Newark, NJ 07013
  Phone: 9739725633

- **Protocol Version and Date:**
  Version 6, 4/8/2021
  Pro#2020001469

- **Co-Investigators:**

  - Steven Libutti, MD
  - Anil Nanda, MD
  - Anne Sutherland, MD
  - Ana Natale, MD
  - Sunil Patel, MD
  - Yingda Xie, MD
  - Dorothy Castro, MD
  - Diana Finkel, MD
  - Amee Patrawala, MD
  - Maninder Pal Kaur, MD
  - Sanna Bdiiwi, MD
  - Nipun Suri, MD
  - Ashish Sonig, MD
  - Priyank Khandelwal, MD
  - James Liu, MD
  - Htay Htay Kyi, MD
  - Hala Boktor, MD
  - Joseph Koziol, MD
  - Patricia Greenberg, MS
  - Nicholas Dadario,

- Rachel Yan
- Kevin Ly
- Sara Subanna

Table of Contents

Skip To Section: Hold **CTRL** + **Click (Below)** To Follow Link in **Blue**

**1.0**	Research Design
1.1	Purpose/Specific Aims
1.2	Research Significance
1.3	Research Design and Methods
1.4	Preliminary Data
1.5	Sample Size Justification
1.6	Study Variables
1.7	Drugs/Devices/Biologics
1.8	Specimen Collection
1.9	Data Collection
1.10	Timetable/Schedule of Events
**2.0**	Project Management
2.1	Research Staff and Qualifications
2.2	Research Staff Training
2.3	Resources Available
2.4	Research Sites
**3.0**	Multi-Center Research
**4.0**	Subject Considerations
4.1	Subject Selection and Enrollment Considerations
4.2	Secondary Subjects
4.3	Number of Subjects
4.4	Consent Procedures
4.5	Special Consent Populations
4.6	Economic Burden and/or Compensation For Subjects
4.7	Risks of Harm/Potential for Benefits to Subjects to Subjects
**5.0**	Special Considerations
5.1	Health Insurance Portability and Accountability Act (HIPAA)
5.2	Family Educational Rights and Privacy Act (FERPA)
5.3	Code of Federal Regulations Title 45 Part 46 (Vulnerable Populations)
5.4	General Data Protection Regulation (GDPR)
5.5	NJ Access to Medical Research Act (Surrogate Consent)
**6.0**	Data Management Plan
6.1	Data Analysis
6.2	Data Security
6.3	Data Safety And Monitoring
6.4	Reporting Results
6.5	Secondary Use of the Data
**7.0**	Research Repositories – Specimens and/or Data
**8.0**	Approvals/Authorizations
**9.0**	Bibliography

1.0 Research Design

1.1 Purpose/Specific Aims

The purpose of this study is to explore the efficacy of Aggrenox (Dipyridamole ER 200mg and Aspirin 25mg), in brand or generic formulation, in patients with SARS-CoV-2 infection with symptoms consistent with COVID-19. For ease, we will identify the drug as Aggrenox, regardless of the formulation.

Among 132 SARS-CoV-2 patients (66 patients in each randomized arm), we will determine the efficacy of Aggrenox on clinical outcomes.

A. Endpoints:

Primary Endpoint:

Change in composite COVID ordinal scale at day 15. Ordinal scale: 1) not hospitalized with resumption of normal activities; 2) not hospitalized, but unable to resume normal activities; 3) hospitalized, not requiring oxygen; 4) hospitalized, requiring oxygen; 5) hospitalized, requiring high-flow oxygen therapy, or noninvasive ventilation; 6) hospitalized, requiring invasive ventilation; 7) ventilation plus additional organ support such as vasopressors, renal replacement therapy and ECMO and 8) death.

Secondary Endpoints:

1) all-cause mortality assessed on day 15 and day 28.
2) oxygen-free days through day 28.
3) invasive-ventilator-free days through day 28.
4) ICU-free days through day 28.
5) hospital-free days through day 28.
6) increase in cycle threshold (Ct) of SARS-CoV-2 PCR test as an approximation of decline in viral load at days 7 and 15.
7) decrease in the markers D-dimer/ ferritin/ c-reactive protein at days 7 and 15.
8) thromboembolic complications including stroke at day 28.
9) Change in COVID ordinal scale at day 28.
10) When feasible urine will be analyzed for markers of tubular injury IL-18, kidney injury molecule-1 [KIM-1], neutrophil gelatinase-associated lipocalin [NGAL]), inflammation (monocyte chemoattractant protein-1 [MCP-1]), and repair (human cartilage glycoprotein-40 [YKL-40]

B. Hypotheses / Research Question(s)

Compared to standard care, addition of Aggrenox (Dipyridamole ER 200mg/ Aspirin 25mg), to standard care will result in improvement in composite COVID ordinal scale at day 15. Additionally, combined Aggrenox (Dipyridamole ER 200mg/ Aspirin 25mg orally/enterally), and standard care will reduce need for ventilation, length of mechanical ventilation, hospital length of stay, ICU length of stay and improve survival more than standard care alone in SARS-CoV-2 patients.

1.2 Research Significance

Coronavirus disease 2019 (COVID-19) is caused by severe acute respiratory syndrome coronavirus 2 (SARS-CoV-2) and which has recently evolved into a pandemic posing a serious

threat to public health worldwide.[1,2]. Thus, development of effective preventative and therapeutic interventions is paramount.

Despite its importance, information about pathologic mechanisms of SARS-CoV-2 remains incomplete. Recent data suggests the role of exaggerated immune/inflammatory response and hypercoagulability as pathogenic mechanisms leading to clinical manifestations such as acute respiratory distress syndrome (ARDS), stroke and extrapulmonary multiorgan dysfunction.[3] Due to the significant activation of coagulation system, the coagulopathy associated with COVID-19 may result in thromboembolic complications such as deep venous thrombosis, pulmonary embolism and strokes.[4]

Prophylactic anti-coagulation therapies have become the standard of care in the management of these patients.[4] The International society of thrombosis and hemostasis has recommended that all COVID-19 patients admitted to the hospital, irrespective of the severity of their illness, should be started on prophylactic dose of low molecular weight heparin (LMWH).[5] The Society of thrombosis and haemostasis research also recommended that all hospitalized COVID-19 patients should receive pharmacological venous thromboembolism (VTE) prophylaxis unless contraindicated.[6] Recent literature shows that the risk of thromboembolism is higher in patients admitted to the ICU. For the patients admitted to the ICU or having additional risk factors for VTE such as BMI> 30mkg/m2, history of VTE or rapidly rising D-dimer levels, high-risk prophylactic LMWH dosages twice daily should be considered.[6] The *therapeutic* doses of either UFH or LMWH should be considered for those who have confirmed thrombosis including filter thrombosis.[7] The anti-inflammatory properties of LMWH have been suggested to provide additional benefits in COVID-19 patients.[4]

Utilization of a therapeutic that can simultaneously intervene along multiple pathways (viral replication, inflammation, vasoconstriction, and coagulation) can dramatically improve patient outcomes.

Adenosine is a highly potent immunoregulatory nucleoside that interferes with inflammatory processes by modulating the biosynthesis and release of proinflammatory cytokines, slowing down the oxidative activity, preventing platelet aggregation, and reducing neutrophils adhesion and degranulation.[8,9] One mechanism to increase extracellular adenosine is to inhibit intracellular uptake of adenosine and thereby capitalize on its anti-inflammatory properties. It has a wide-ranging effects on multiple organ systems by interacting with different adenosine receptors.[9] Equilibrative nucleoside transporters (ENTs) are nucleoside transporters that regulate the flux of endogenous nucleosides, nucleobases and therapeutic analogs.[10] Given the critical nature of regulating nucleosides in a number of pathological processes, ENTs have emerged as a druggable target. ENT inhibitors are used to modulate nucleosides in a number of disease states including viral infections such as HIV[11], and tumors[12].

Dipyridamole (DIP) is one such ENT inhibitor (specific for the major and ubiquitous nucleoside transporter ENT-1), that inhibits nucleoside (adenosine) uptake into the endothelial and other cells and has been shown to *enhance the anti-inflammatory and immunomodulatory effect of adenosine*.[13] A recent study has shown that DIP increases extracellular adenosine levels and thereby provides a significant decrease in CD8+T-cell activation and decreased macrophage activation among persons with HIV-1 infection on antiviral therapy.[11]

DIP may have additional therapeutic advantages to SARS-CoV-2 patients. DIP is a widely known *phosphodiesterase (PDE) inhibitor with antiplatelet*[14] *and vasodilatoratory*[15] *effects*. Of particular note, a recent screen of FDA approved drugs identified the ability of DIP to *effectively suppress SARS-CoV-2 replication* with a therapeutically achievable EC50 concentration of 100

nM.[16] Moreover, a small study of 22 COVID-19 patients suggested improved outcomes including reduction in coagulation markers.[17] These antiviral data are not without precedent as DIP has also been shown to have *powerful antiviral activity against single-stranded RNA viruses* in vitro, and in a VSV-induced viral pneumonia model in vivo.[18-20] Additional possible benefits could include the anti-inflammatory action and promotion of mucosal healing.[21] It may also prevent acute injury and progressive fibrosis of the lung, heart, liver, and kidney.[22] In one small clinical series of 12 patients (pre-print, not peer-reviewed data), the authors used DIP as a prophylactic anti-coagulation therapy and reported significantly increased platelet and lymphocyte counts and decreased D-dimer levels in comparison to control patients. Two weeks after initiation of DIP therapy 60% of their severe patients and all patients with mild disease were discharged from the hospital.[17]

Aspirin is a well-known anti-platelet drug with anti-inflammatory properties. Aspirin inhibits platelet cyclooxygenase-1 activity that is essential for generation of thromboxane A2. Thromboxane A2 is a potent vasoconstrictor that also induces platelet aggregation and release of proinflammatory proteins from platelet storage granules. The literature evidence suggests that aspirin plus extended-release dipyridamole may be more suitable for preventing secondary stroke because, in addition to their antiplatelet actions, these drugs possess complementary anti-inflammatory properties.[23]

Thus, we hypothesize that Aggrenox (Dipyridamole ER 200mg/ Aspirin 25mg orally/enterally), adjunctive therapy can be useful for SARS-CoV-2 infected patients through enhancing immune recovery, antiviral properties, and correcting the coagulation profile.

We propose a randomized, 2-arm, open-label single-site pilot study to evaluate the effect of oral/enteral Aggrenox on clinical outcomes in patients with SARS-CoV. We will randomly assign 132 consenting participants to two treatment groups: 1) Aggrenox (Dipyridamole ER 200mg/ Aspirin 25mg orally/enterally), + standard care and 2) standard care alone. We will collect demographic, clinical, laboratory and radiological data. The patients would be followed daily for 2 weeks after enrollment while the patient is in the hospital and once discharged, they will be called every 3rd day to follow up on the symptoms.

1.2 Research Design and Methods

1: 1 Randomized design.

Arm 1: Aggrenox plus standard care. Participants will receive Aggrenox (Dipyridamole ER 200mg/ Aspirin 25mg orally/enterally), 2 times daily (FDA–recommended dose) starting on the day of enrollment for a total of 2 weeks along with standard care.
Arm 2: Standard care. Participants will receive standard care starting on the day of enrollment for a total of 2 weeks.

We will perform a randomized, 2-arm, open-label single-site pilot study to evaluate the effect of oral Aggrenox (Dipyridamole ER 200mg/ Aspirin 25mg orally/enterally), on clinical outcomes in patients with SARS-CoV-2. In this research proposal, we will randomly assign 132 consenting participants with diagnosis of SARS-CoV-2 to two treatment groups: 1) Aggrenox (Dipyridamole ER 200mg/ Aspirin 25mg orally/enterally) + standard care and 2) standard care alone. Participants will be screened, enrolled, receive treatment and followed for 28 days. The study aim and procedure will be explained to every eligible subject and informed consent will be obtained from interested subjects or authorized proxy to participate in the study. We do not

anticipate problems with enrollment as sufficient number of COVID-19 patients are admitted at Rutgers-NJMS (approximately 2-5 new COVID-19 admissions daily, and total 18-20 patients who are currently admitted at Rutgers-NJMS with 5-6 patients in intensive care units).

Randomization. The P.I. or study coordinator will review the screening and enroll participants in the randomization. Once a participant has been randomized, we will conduct all evaluations irrespective of whether the participant starts the treatment, and how long the participants continues on the treatment or not. SARS-CoV-2 patients will be allocated in a 1:1 randomized fashion using computer generated random sequence to one of two groups: the Aggrenox (Dipyridamole ER 200mg/ Aspirin 25mg orally/enterally), plus standard care or the standard care. Data collection will end after 28 days, or the patient dies or withdraws consent.

A. Research Procedures

Study Procedures:

Day 0 – Screening Visit: (Complete baseline COVID Case report form – Appendix B: Module 1)

o Review eligibility criteria
o Obtain informed consent for trial enrollment
o Demographic data
o Medical history
o Physical examination – will be performed as part of the routine care and will be abstracted from the medical record
o Vital signs (blood pressure, heart rate, temperature) - will be abstracted from the medical record
o Electrocardiogram - will be performed as part of the routine care and will be abstracted from the medical record
o Labs: CBC, BMP, Coagulation profile, Ferritin, D-Dimer, CRP. will be collected as part of the routine care and will be abstracted from the medical record
o Concomitant medications (starting from the time the patient signs the informed consent)
o Collection of research samples:

- Saliva, oral, nasal, and pharyngeal swabs, and sputum (if spontaneously producing sputum or intubated) for SARS-CoV-2 RNA will be collected at baseline day 0

o Randomize study participant through RedCap.

Treatment Period –Days 1 - 14 (Complete daily COVID Case report form – Appendix B: Module 2)

▪ **Clinical assessment** completed by Research Nurse. May be conducted by phone if the patient is discharged before 14 days.

- Review of daily temperatures
- Review of medication compliance
- Adverse events
- Record standard of care treatments and/or procedures prescribed

- **Labs:** At day 7 and day 14: we will abstract CBC, BMP, Coagulation profile, Ferritin, D-Dimer, CRP results from the medical record.

- **Collect research specimens**:
    - Saliva, oral, nasal, and pharyngeal swabs, and sputum (if spontaneously producing sputum or intubated) for SARS-CoV-2 RNA will be collected at day 7 and day 14 in a subset of patients.
    - Urine samples will be collected on day 1 and day 14.

Treatment Period –Days 15 - 28 **(**Complete daily COVID Case report form – Appendix B: Module 2)

▪ **Clinical assessment** completed by Research Nurse. May be conducted by phone if the patient is discharged before 14 days.

- Review of daily temperatures
- Adverse events
- Record standard of care treatments and/or procedures prescribed
- COVID ordinal outcomes scale on day 28.

Discharge/ Death: Complete COVID Case report form –(Appendix B: Module 3)

Treatment Plan:

Participants will be randomized 1:1 to Aggrenox or standard treatment.

- Active Comparator: (Aggrenox (Dipyridamole ER 200mg/ Aspirin 25mg orally/enterally). Participants will receive Aggrenox (Dipyridamole ER 200mg/ Aspirin 25mg orally/enterally), 2 times daily (FDA–recommended dose) starting on the day of enrollment for a total of 2 weeks + standard care.
- Standard care Comparator: Participants will receive standard care starting on the day of enrollment for a total of 2 weeks.

2. Subject Enrollment

1. Patients who are eligible for the study will be enrolled in the clinical trial after they or their authorized proxy provide informed consent for study participation. A copy of the institution's IRB approved informed consent will be on file RedCap. The patients will be enrolled through RedCap, the Clinical Trials Management System for this study. Contact the Rutgers Clinical Trials Unit, if you have any questions about the Registration/Enrollment process.

• Registration: Any subject that has signed the consent will be entered into RedCap. A Copy of the consent will be uploaded into the Documents section.

• Enrollment: Once eligibility has been confirmed, the completed, signed and dated eligibility checklist will be uploaded into the Documents section. RedCap will randomize the patient and a sequence number (subject study ID) will be generated at the time of enrollment, this is the point the patient is considered on study.

Patients will not start protocol treatment prior to registration.

3. Study Safety Assessments

The Principal Investigator is responsible for ensuring that all staff involved in the study are familiar with the content of this section.

Definitions:

• Adverse Events:

An adverse event is the development of an undesirable medical condition or the deterioration of a pre-existing medical condition following or during exposure to a pharmaceutical product, whether considered causally related to the product. An undesirable medical condition can be symptoms (e.g. nausea, chest pain), signs (e.g. tachycardia, enlarged liver) or the abnormal results of an investigation (e.g. laboratory findings, electrocardiogram). In clinical studies, an AE can include an undesirable medical condition occurring at any time, including run-in or washout periods, even if no study treatment has been administered. The term AE is used to include both serious and non- serious AEs.

• Serious Adverse Events:

A serious adverse event is an AE occurring during any study phase (i.e., screening, run- in, treatment, wash-out, follow-up), at any dose of the study drugs that fulfills one or more of the following criteria and deemed secondary to the use of study drug:

- ➢ Results in death
- ➢ Is immediately life-threatening
- ➢ Requires in-patient hospitalization or prolongation of existing hospitalization
- ➢ Results in persistent or significant disability or incapacity
- ➢ Is a congenital abnormality or birth defect
- ➢ Is an important medical event that may jeopardize the patient or may require medical intervention to prevent one of the outcomes listed above.

1.1.1 The causality of SAEs (their relationship to all study treatment/procedures) will be assessed by the investigator(s) and communicated to Boehringer Ingelheim (BI) via BI SAE form and the FDA via MedWatch form, if applicable.

Causality will be reported as either "Yes" or "No".

- ➢ Yes: There is a reasonable causal relationship between the investigational product administered and the AE.
- ➢ No: There is no reasonable causal relationship between the investigational product administered and the AE.

2. Recording of Adverse Events

Non-serious adverse events and SAEs will be determined from the time the patient is enrolled into the study and up to and including the 2 weeks follow-up period (after the completion of 2 weeks of treatment with Aggrenox) for a total of 28 days . All grade 3 or higher AEs will be

recorded. Grade 1-2 AEs do not need to be recorded. After withdrawal from treatment, subjects must be followed-up for all existing and new AEs for 14 calendar days after the last dose of trial drug and/or until event resolution. All new AEs occurring during that period must be recorded (if SAEs, they must be reported to the DSMB and Rutgers IRB as per SOPs.). AEs will be recorded per Rutgers IRB SOPs. The common terminology criteria for adverse events (CTCAE) will be used as a reference to grade the adverse events. (https://ctep.cancer.gov/protocoldevelopment/electronic_applications/docs/CTCAE_v5_Quick_Reference_8.5x11.pdf (Accessed on March 09, 2018)

All study-related adverse events/ SAEs must be followed until resolution, unless in the Treating Investigator's opinion, the condition is unlikely to resolve due to the patient's underlying disease.

3. Reporting of Serious Adverse Events

Investigators and other site personnel must inform the Rutgers IRB per the SOPs on the required forms. FDA will be informed per SOP, via a MedWatch, of any serious or unexpected adverse events that occur in accordance with the reporting obligations of 21 CFR 312.32. It is the responsibility of the principal investigator to compile all necessary information and ensure that the FDA receives a report according to the FDA reporting requirement timelines.

In addition to FDA, the Sponsor shall report all SAEs and non-serious AEs that occur ,throughout the treatment period and up to and including the 2 weeks follow-up period (after the completion of 2 weeks of treatment with Aggrenox) for a total of 28 days which are relevant for a reported SAEs and any AEs relevant to a reported SAE by fax or other secure method using BI IIS SAE form to the BI Unique Entry Point in accordance with timeline specified in the safety data exchange agreement .

For each adverse event, the investigator will provide the onset date, end date, causality, intensity,treatment required, outcome, seriousness, and action taken with the investigational drug.

The investigator does not need to actively monitor patients for adverse events once the clinical trial has ended. However, if the investigator becomes aware of an SAE(s) that occurred after the patient has completed the clinical trial (including any protocol specified follow-up period), it should be reported to BI if investigator considers it as relevant to the BI study drug.

All SAEs meeting the criteria for expedited reporting will be reported to the Rutgers IRB within the mandated time frames. All SAEs within the safety follow-up window (e.g., within 14 days after the last dose of study medications) established in the protocol will be reported.

Non-serious adverse events and SAEs will be collected from the time the patient is enrolled into the study, throughout the treatment period and up to and including the 2 weeks follow-up period (after the completion of 2 weeks of treatment with Aggrenox) for a total of 28 days. After withdrawal from treatment, subjects must be followed-up for all existing and new AEs for 14 calendar days after the last dose of trial drug and/or until event resolution. All new AEs occurring during that period must be recorded (if SAEs, then they must be reported to the FDA as per their reporting criteria). All study-related toxicities/ SAEs must be followed until resolution, unless in the Treating Investigator's opinion, the condition is unlikely to resolve due to the patient's underlying disease.

Clinical Safety Assessments

- <u>Recording of Adverse Events</u>

Non-serious adverse events and SAEs will be determined from the time the study drug is given, throughout the treatment period and up to and including the 2 weeks follow up period. All grade 3 or higher AEs will be recorded. Grade 1-2 AEs do not need to be recorded. After withdrawal from treatment, subjects must be followed-up for all existing and new AEs for 14 calendar days after the last dose of trial drug and/or until event resolution. All new AEs occurring during that period must be recorded (if SAEs, they must be reported to the DSMB and Rutgers IRB as per SOPs.). AEs will be recorded per Rutgers IRB SOPs.

- Adverse Events Based on Signs and Symptoms

When collecting AEs, the recording of diagnoses is preferred (when possible) to recording a list of signs and symptoms. However, if a diagnosis is known and there are other signs or symptoms that are not generally part of the diagnosis, the diagnosis and each sign or symptom will be recorded separately.

- Vital Signs

Temperature will be obtained at least two times a day (AM and PM). Nursing staff will record the temperature while the patient is in the hospital. Body temperature will be measured in degrees Fahrenheit using an automated thermometer. An automated thermometer will be provided to the patient once discharged from the hospital for checking the temperature at least once a day for the study duration (28 days total from the enrollment day).

Study Efficacy/Response Assessments

Change in composite COVID ordinal scale at day 15. Ordinal scale: 1) not hospitalized with resumption of normal activities; 2) not hospitalized, but unable to resume normal activities; 3) hospitalized, not requiring oxygen; 4) hospitalized, requiring oxygen; 5) hospitalized, requiring high-flow oxygen therapy, or noninvasive ventilation; 6) hospitalized, requiring invasive ventilation; 7) ventilation plus additional organ support such as pressors, renal replacement therapy and ECMO and 8) death.

Other response criteria used as secondary endpoints include the need of invasive ventilation, the days dependent on ventilator, hospital free days, thromboembolic complications, COVID ordinal scale at day 28 and mortality.

Data Points

Case Report Forms[24]

Completion of the electronic case report forms (eCRFs) will be done in accordance with the instructions outlined in the study specific data capture plan. All eCRFs are considered the primary data collection document for the study and are stored in RedCap in a confidential format. Only key personnel who are delegated in the delegation of authority log are permitted to make entries, changes, or corrections in the eCRF. All users of RedCap will complete user training, as required or appropriate per regulations. An audit trail will be maintained automatically by the electronic CRF management system.

<u>Data Points to be collected:</u>

- Baseline medical history
- Treatment compliance
- Daily temperatures
- Symptoms of disease
    - Adverse events
    - Concomitant medications
    - Any standard of care treatments or procedures to manage COVID-19

Data Submission Timeline and Forms
Completion of eCRFs will occur within 72 hours of study time point unless otherwise indicated. Baseline (pre-study) eCRFs (e.g., enrollment, medical history, concomitant medications, etc.) will be completed no later than 24 hours after the start of treatment.

Study Duration: Study participants will be treated for fourteen (14) days and will then remain on follow-up for up to 4 weeks (28 days) total from their enrollment. The study will end for each subject upon the completion of their day 28 post enrollment follow-up interview.

The study will be conducted over approximately 24 months: 6-8 months for enrollment and follow-up/data collection and 18 months for specimen processing and analysis, and data analysis.

We will account for all the patients registered in the study. The number of patients who were not evaluable, who died or withdrew before treatment was completed will be specified. The distribution of follow-up time will be described and the number of patients lost to follow-up will be given.

B. Endpoints

Primary endpoint:

Change (as compared to baseline on day 0) in composite COVID ordinal scale at day 15 Ordinal scale: 1) not hospitalized with resumption of normal activities; 2) not hospitalized, but unable to resume normal activities; 3) hospitalized, not requiring oxygen; 4) hospitalized, requiring oxygen; 5) hospitalized, requiring high-flow oxygen therapy, or noninvasive ventilation; 6) hospitalized, requiring invasive ventilation; 7) ventilation plus additional organ support such as vasopressors, renal replacement therapy and ECMO and 8) death.

Secondary endpoints:

1) all-cause mortality assessed on day 15 and day 28.
2) oxygen-free days through day 28.
3) invasive-ventilator-free days through day 28.
4) ICU-free days through day 28.
5) hospital-free days through day 28.
6) increase in cycle threshold (Ct) of SARS-CoV-2 PCR test as an approximation of decline in viral load at days 7 and 15.
7) Change in the inflammatory markers D-dimer/ ferritin/ c-reactive protein at days 7 and 15 as compared to baseline on day 0.
8) thromboembolic complications including stroke at day 28.

9) Change (as compared to baseline on day 0) in COVID ordinal scale at day 28.
10) if feasible urine will be analysed for markers of tubular injury IL-18, kidney injury molecule-1 [KIM-1], neutrophil gelatinase-associated lipocalin [NGAL]), inflammation (monocyte chemoattractant protein-1 [MCP-1]), and repair (human cartilage glycoprotein-40 [YKL-40]

1.4 **Preliminary Data**
This is the first study of its kind. No preliminary data is available with the use of Aggrenox in patients with COVID-19. The use of Dipyridamole in a small unpublished study from China showed better outcomes in patients with COVID-19.[17]

1.5 Sample Size Justification
The sample size of 132 patients (66 patients in each arm) was estimated to provide 90% power to detect a relative between-group difference of 20% reduction in composite ordinal scale (from day 0 to day 15) with assumption of mean composite ordinal scale value of 4.5 ± 1.6 (yes- this is SD) at baseline (day 0) and two-sided P value of 0.05.[25] For primary efficacy, we will do an intention-to-treat analysis and include all patients who have undergone randomization and will adjust for the minimization variables. We will analyze continuous measures by means of linear regression and present as mean differences. For binary outcomes, we will present data as risk ratios from a binomial regression model.

1.6 Study Variables

A. **Independent Variables, Interventions, or Predictor Variables**
The following variables will be examined for treatment outcomes of efficacy and/or predict outcomes:

- Treatment
- Baseline temperature
- Baseline SARS-CoV test
- Baseline measure of inflammatory markers

B. **Dependent Variables or Outcome Measures**
The following variables will be examined for changed as result of treatment or as predictors

- Treatment Compliance
- Change in the fever curve
- Improvement in vital signs
- Time to discharge (if hospitalized)
- Adverse Events (number/percentage/grade)
- Inpatient versus Outpatient
- Change from baseline SARS-CoV test
- Change from baseline inflammatory markers

1.**7 Drugs/Devices/Biologics**

Generic name: Dipyridamole and Aspirin

Commercial name: Aggrenox

Aggrenox is a combination antiplatelet agent intended for oral administration. Each hard gelatin capsule contains 200 mg dipyridamole in an extended-release form and 25 mg aspirin, as an immediate- release sugar-coated tablet. In addition, each capsule contains the following inactive ingredients: acacia, aluminum stearate, colloidal silicon dioxide, corn starch, dimethicone, hypromellose, hypromellose phthalate, lactose monohydrate, methacrylic acid copolymer, microcrystalline cellulose, povidone, stearic acid, sucrose, talc, tartaric acid, titanium dioxide, and triacetin. Each capsule shell contains gelatin, red iron oxide and yellow iron oxide, titanium dioxide, and water.

Source: Commercially available

For complete information please refer to the package inserts at
https://dailymed.nlm.nih.gov/dailymed/search.cfm?labeltype=all&query=aggrenox

Dipyridamole
Dipyridamole is an antiplatelet agent chemically described as 2,2',2",2'"-[(4,8-Dipiperidinopyrimido[5,4-d]pyrimidine-2,6-diyl)dinitrilo]-tetraethanol. It has the following structural formula:

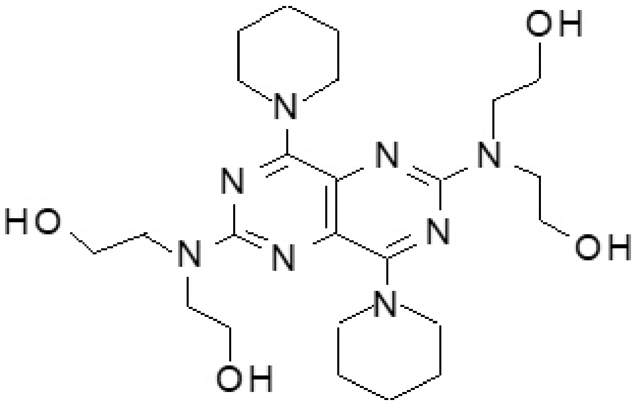

$C_{24}H_{40}N_{8}O_{4}$ Mol. Wt. 504.63
Dipyridamole is an odorless yellow crystalline substance, having a bitter taste. It is soluble in dilute acids, methanol and chloroform, and is practically insoluble in water.

Aspirin
The antiplatelet agent aspirin (acetylsalicylic acid) is chemically known as benzoic acid, 2-(acetyloxy)-, and has the following structural formula:
$C_{9}H_{8}O_{4}$ Mol. Wt. 180.16
Aspirin is an odorless white needle-like crystalline or powdery substance. When exposed to moisture, aspirin hydrolyzes into salicylic and acetic acids, and gives off a vinegary odor. It is highly lipid soluble and slightly soluble in water.
12 CLINICAL PHARMACOLOGY 12.1 Mechanism of Action

The antithrombotic action of AGGRENOX is the result of the additive antiplatelet effects of dipyridamole and aspirin.

Dipyridamole
Dipyridamole inhibits the uptake of adenosine into platelets, endothelial cells and erythrocytes in vitro and in vivo; the inhibition occurs in a dose-dependent manner at therapeutic concentrations

(0.5–1.9 µg/mL). This inhibition results in an increase in local concentrations of adenosine which acts on the platelet A_2-receptor thereby stimulating platelet adenylate cyclase and increasing platelet cyclic-3',5'- adenosine monophosphate (cAMP) levels. Via this mechanism, platelet aggregation is inhibited in response to various stimuli such as platelet activating factor (PAF), collagen and adenosine diphosphate (ADP).
Dipyridamole inhibits phosphodiesterase (PDE) in various tissues. While the inhibition of cAMP-PDE

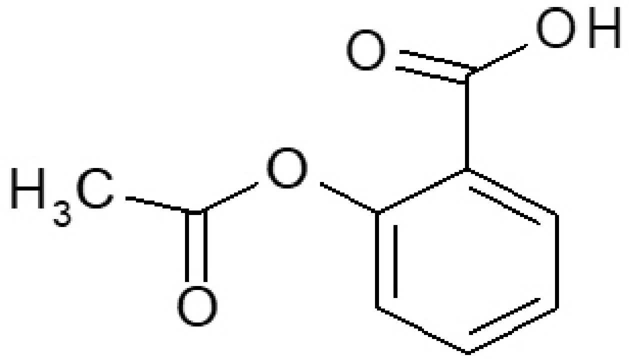

is weak, therapeutic levels of dipyridamole inhibit cyclic-3',5'-guanosine monophosphate-PDE (cGMP- PDE), thereby augmenting the increase in cGMP produced by EDRF (endothelium-derived relaxing factor, now identified as nitric oxide).

Aspirin
Aspirin inhibits platelet aggregation by irreversible inhibition of platelet cyclooxygenase and thus inhibits the generation of thromboxane A_2, a powerful inducer of platelet aggregation and vasoconstriction.

Pharmacodynamics
The effect of either agent on the other's inhibition of platelet reactivity has not been evaluated.

Pharmacokinetics
There are no significant interactions between aspirin and dipyridamole. The kinetics of the components are unchanged by their co-administration as AGGRENOX.
Absorption

Dipyridamole:
Peak plasma levels of dipyridamole are achieved 2 hours (range 1–6 hours) after administration of a daily dose of 400 mg AGGRENOX (given as 200 mg BID). The peak plasma concentration at steady- state is 1.98 µg/mL (1.01–3.99 µg/mL) and the steady-state trough concentration is 0.53 µg/mL (0.18– 1.01 µg/mL).

Aspirin:
Peak plasma levels of aspirin are achieved 0.63 hours (0.5–1 hour) after administration of a 50 mg aspirin daily dose from AGGRENOX (given as 25 mg BID). The peak plasma concentration at steady- state is 319 ng/mL (175–463 ng/mL). Aspirin undergoes moderate hydrolysis to salicylic acid in the liver and the gastrointestinal wall, with 50%–75% of an administered dose reaching the systemic circulation as intact aspirin.

Effect of Food

Dipyridamole:
When AGGRENOX capsules were taken with a high fat meal, dipyridamole peak plasma levels (C_{max}) and total absorption (AUC) were decreased at steady-state by 20-30% compared to fasting. Due to the similar degree of inhibition of adenosine uptake at these plasma concentrations, this food effect is not considered clinically relevant.

Aspirin:
When AGGRENOX capsules were taken with a high fat meal, there was no difference for aspirin in AUC at steady-state, and the approximately 50% decrease in C_{max} was not considered clinically relevant based on a similar degree of cyclooxygenase inhibition comparing the fed and fasted state.

Distribution

Dipyridamole:
Dipyridamole is highly lipophilic (log P=3.71, pH=7); however, it has been shown that the drug does not cross the blood-brain barrier to any significant extent in animals. The steady-state volume of distribution of dipyridamole is about 92 L. Approximately 99% of dipyridamole is bound to plasma proteins, predominantly to alpha 1-acid glycoprotein and albumin.

Aspirin:
Aspirin is poorly bound to plasma proteins and its apparent volume of distribution is low (10 L). Its metabolite, salicylic acid, is highly bound to plasma proteins, but its binding is concentration-dependent (nonlinear). At low concentrations (<100 mcg/mL), approximately 90% of salicylic acid is bound to albumin. Salicylic acid is widely distributed to all tissues and fluids in the body, including the central nervous system, breast milk, and fetal tissues. Early signs of salicylate overdose (salicylism), including tinnitus (ringing in the ears), occur at plasma concentrations approximating 200 mcg/mL.

Metabolism and Elimination

Dipyridamole:
Dipyridamole is metabolized in the liver, primarily by conjugation with glucuronic acid, of which monoglucuronide which has low pharmacodynamic activity is the primary metabolite. In plasma, about 80% of the total amount is present as parent compound and 20% as monoglucuronide. Most of the glucuronide metabolite (about 95%) is excreted via bile into the feces, with some evidence of enterohepatic circulation. Renal excretion of parent compound is negligible and urinary excretion of the glucuronide metabolite is low (about 5%). With intravenous (i.v.) treatment of dipyridamole, a triphasic profile is obtained: a rapid alpha phase, with a half-life of about 3.4 minutes, a beta phase, with a half- life of about 39 minutes, (which, together with the alpha phase accounts for about 70% of the total area under the curve, AUC) and a prolonged elimination phase λ_z with a half-life of about 15.5 hours. Because of the extended absorption phase of the dipyridamole component, only the terminal phase is apparent from oral treatment with AGGRENOX which was 13.6 hours.

Aspirin:

Aspirin is rapidly hydrolyzed in plasma to salicylic acid, with a half-life of 20 minutes. Plasma levels of aspirin are essentially undetectable 2–2.5 hours after dosing and peak salicylic acid concentrations occur 1 hour (range: 0.5–2 hours) after administration of aspirin. Salicylic acid is primarily conjugated in the liver to form salicyluric acid, a phenolic glucuronide, an acyl glucuronide, and a number of minor metabolites. Salicylate metabolism is saturable and total body clearance decreases at higher serum concentrations due to the limited ability of the liver to form both salicyluric acid and phenolic glucuronide. Following toxic doses (10–20 g), the plasma half-life may be increased to over 20 hours.

The elimination of acetylsalicylic acid follows first-order kinetics with AGGRENOX and has a half- life of 0.33 hours. The half-life of salicylic acid is 1.71 hours. Both values correspond well with data from the literature at lower doses which state a resultant half-life of approximately 2–3 hours. At higher doses, the elimination of salicylic acid follows zero-order kinetics (i.e., the rate of elimination is constant in relation to plasma concentration), with an apparent half-life of 6 hours or higher. Renal excretion of unchanged drug depends upon urinary pH. As urinary pH rises above 6.5, the renal clearance of free salicylate increases from <5% to >80%. Alkalinization of the urine is a key concept in the management of salicylate overdose [see Overdosage (10)]. Following therapeutic doses, about 10% is excreted as salicylic acid and 75% as salicyluric acid, as the phenolic and acyl glucuronides, in urine.

Specific Populations
Geriatric Patients:

Dipyridamole:
In ESPS2 [see Clinical Studies (14)], plasma concentrations (determined as AUC) of dipyridamole in healthy elderly subjects (>65 years) were about 40% higher than in subjects younger than 55 years receiving treatment with AGGRENOX.

Hepatic Dysfunction:
No study has been conducted with AGGRENOX in patients with hepatic dysfunction.

Dipyridamole:
In a study conducted with an intravenous formulation of dipyridamole, patients with mild to severe hepatic insufficiency showed no change in plasma concentrations of dipyridamole but showed an increase in the pharmacologically inactive monoglucuronide metabolite. Dipyridamole can be dosed without restriction as long as there is no evidence of hepatic failure.

Aspirin:
Avoid aspirin in patients with severe hepatic insufficiency.

Renal Dysfunction:

Dipyridamole:
In ESPS2 patients [see Clinical Studies (14)], with creatinine clearances ranging from about 15 mL/min to >100 mL/min, no changes were observed in the pharmacokinetics of dipyridamole or its glucuronide metabolite if data were corrected for differences in age.

Aspirin:
Avoid aspirin in patients with severe renal failure (glomerular filtration rate <10 mL/min). Drug Interaction Studies

A dedicated drug interaction study was conducted in 60 healthy volunteers to evaluate the effects of omeprazole 80 mg administered once daily on the pharmacokinetics (PK) of dipyridamole and the pharmacodynamics (PD) of acetylsalicylic acid when co-administered with AGGRENOX twice daily. Dipyridamole exposure (C_{max} and AUC) at steady-state were similar with or without omeprazole co- administration. The pharmacokinetics of acetylsalicylic acid was not characterized. However, the antiplatelet activity as measured by arachidonic acid induced platelet aggregation was similar between the treatment arms at steady-state.

NONCLINICAL TOXICOLOGY

Carcinogenesis, Mutagenesis, Impairment of Fertility
In studies in which dipyridamole was administered in the feed to mice (up to 111 weeks in males and females) and rats (up to 128 weeks in males and up to 142 weeks in females), there was no evidence of drug-related carcinogenesis. The highest dose administered in these studies (75 mg/kg/day) was, on a 2 mg/m basis, about equivalent to the maximum recommended daily human oral dose(MRHD)in mice and about twice the MRHD in rats.

Combinations of dipyridamole and aspirin (1:5 ratio) tested negative in the Ames test, in vivo chromosome aberration tests (in mice and hamsters), oral micronucleus tests (in mice and hamsters) and oral dominant lethal test (in mice). Aspirin, alone, induced chromosome aberrations in cultured human fibroblasts. Mutagenicity tests of dipyridamole alone with bacterial and mammalian cell systems were negative.

Combinations of dipyridamole and aspirin have not been evaluated for effects on fertility and reproductive performance. There was no evidence of impaired fertility when dipyridamole was administered to male and female rats at oral doses up to 500 mg/kg/day (about 12 times the MRHD on a mg/m2 basis). A significant reduction in number of corpora lutea with consequent reduction in implantations and live fetuses was, however, observed at 1250 mg/kg (more than 30 times the MRHD on a mg/m2 basis). Aspirin inhibits ovulation in rats.

A. Drug/Device Accountability and Storage Methods
Drug accountability logs and pharmacy records must be maintained at the hospital pharmacy and must be available for review upon request. Drug accountability logs and pharmacy records will be reviewed during monitoring visits and by the Research Pharmacy Shared Resource of Rutgers Cancer Institute of New Jersey.

B. Treatment Compliance
The study medication will be given in accordance with the protocol and the instructions of the treating investigator. For those subjects who will be discharged from the hospital before 14 days, the study drugs will be provided to patients to be self-administered together with the current Aggrenox product information. They will be requested to keep pill diary. During the telephone visits with the patients every 3rd day after discharge, the patients will be enquired about their compliance with the medications. A pill diary will be provided and must be completed by the patient to document treatment course.

1.8 Specimen Collection

Primary Specimen Collection

The research samples collected will be studied using standard laboratory assays for:

1. SARs-CoV-2 RNA

- **Types of Specimens:**
  - Saliva, oral, nasal, and pharyngeal swabs, and sputum (if spontaneously producing sputum or intubated) for SARS-CoV-2 RNA will be collected at baseline day 0, day 7 and day 14 in a subset of patients.
  - Urine samples will be collected on day 1 and day 14.
- **Annotation:** Data to be annotated or associated with each specimen include participant ID (PID), date and time of collection, and specimen type. A PID will be assigned at the time of screening; at the time of specimen collection, all specimens collected will be labelled with the PID, date/time of collection, study timepoint, and specimen type. No identifiers will be included on the labels. These PIDs will be used to link the specimens with REDCap database containing the data collected from the EHR. All identifiers include name and date of birth matched to PID will be kept in a separate encrypted, password-protected electronic file that is accessible only by designated study personnel.
- **Transport:** All samples will be collected into sealed container(s) with de-identified PID-encoded labels and placed in a cooler. Samples will be transported to the hospital hematology and microbiology labs by trained personnel. The respiratory samples (saliva, swabs, sputum) will be processed in the Banada laboratory. Urine specimens will be transported by the study coordinator to a -80C freezer in the MSB A900 unit for temporary storage until analysis.
- **Processing:** Specimen processing will occur in accordance with approved Rutgers NJMS Institutional Biosafety Committee protocols.
- **Storage:** Urine will be stored at -80C in freezers in the MSB until processing and accessible by the study investigators and their laboratory personnel. Respiratory samples will be stored at -80C in the RBL, accessible by Dr. Xie and their laboratory personnel.
- **Disposition:** Analysis will occur at the end of the study/accrual period. Any unused specimens will be discarded upon study completion following appropriate biohazard disposal procedures by Xie laboratory personnel for the respective specimens.

B. Secondary Specimen Collection

N/A

1.9 Data Collection

1. **Primary Data Collection**
   Electronic COVID case report forms (eCRFs)[24] have been designed to collect the data points outlined in section 1.3 (D). Completion of the electronic CRFs (eCRFs) will be done in accordance with the instructions in a study specific data capture plan. All eCRFs will be completed by the research staff of the Clinical research unit. The eCRFs will be maintained in a confidential format in the RedCap database.

2. **Secondary Data Collection**
   N/A

1.10 Timetable/Schedule of Events

Please refer to Appendix A. All study procedures will be performed according to the schedule outlined in the Study Flow Table

2.0 Project Management

2.1 Research Staff and Qualifications

Researchers and research Staff are part of the Rutgers- New Jersey Medical School, and/or Clinical Research Unit (CRU) Rutgers University. The Investigators and staff are qualified by training and experience for their research roles, including knowledge of applicable laws, regulations, codes, guidance, and relevant professional standards.

2.2 Research Staff Training

All research team members will complete the required Human Subject Protection training through the Collaborative Institutional Training Initiative (CITI) Program prior to being approved to take part on this study. All study members will attend the site initiation visit for training about the study specific protocol.

The Principal Investigator will provide oversight and ensure that the study is conducted according to the investigational plan and applicable regulations in accordance with 21 CFR 312.

2.3 Resources Available

Aggrenox Capsule (25 mg aspirin/200 mg extended-release dipyridamole) will be provided at no charge to the study participants.

2.4 Research Sites

University Hospital – 150 Bergen Street

Rutgers New Jersey Medical School:
Doctors Office Center, 90 Bergen Street
Medical Science Building, 185 South Orange Ave

St. Barnabas Medical Center
Livingston Hospital
94 Old Short Hills Rd, Livingston, NJ 07039

3.0 Multi-Center Research

NA

4.0 Subject Considerations

4.1 **Subject Selection and Enrollment Considerations**

1. **Method to Identify Potential Subjects**
   An IRB approved press release will be distributed to Rutgers physicians through the Rutgers Communications Office. Press release will have a phone number for the Rutgers physicians

to call the research team about the hospitalized patient as a potential study participant. Potential study participants will also be identified by the research team members. Screening will be conducted by to be named Advanced Practice Nurse and Research Nurse. All research personnel will wear appropriate PPE as outlined.

2. **Recruitment Details**

The subjects will be adults age 18 years and older with a diagnosis of COVID-19 and a confirmed positive test for SARS-CoV-2 by any accepted method. Potential study participants will be recruited by the research team members. The research team will also review each page of the informed consent document with the patient or authorized proxy. This study will be available to all patients who meet the eligibility criteria. There will be no limitation to access with regards to race or gender. Patients will be required to sign/ verbal consent on an IRB- approved informed consent form prior to commencing any research related activity.

Subject Screening

If the patient lacks decision-making capacity, then authorized proxy will be approached for the consent for the patient to participate in this study. The treating investigator and research team will interview the patient/ proxy and review the chart to determine if the patient is a potential candidate for the study. Screening tests to confirm whether subjects are eligible for inclusion in this study will be conducted including SARS-CoV-2 test if not already confirmed to be positive. The Principal Investigator, or designee, will be responsible for reviewing and confirming subject eligibility prior to enrollment to the study.

Subjects who do not meet all inclusion criteria will be recorded as screen failures in RedCap.

Inclusion Criteria:

1. Age ≥18 years.
2. Hospitalization.
3. SARS-CoV-2 viral nucleic acid positive within 3 days.
4. Lab test result pending plus a high clinical suspicion for SARS-CoV-2 (fever and cough for ≤ 7 days, bilateral pulmonary infiltrates on imaging or new hypoxemia with spO2 ≤94% on room air or no alternative explanation for respiratory symptoms).
5. Willing and able to provide consent or by authorized proxy.

Exclusion Criteria:

1. Pregnancy.
2. History of G-6PD deficiency.
3. Use of antiplatelet agents including inhibitor of P2Y12 ADP platelet receptors, phosphodiesterase inhibitors, and Glycoprotein IIB/IIIA inhibitors.
4. On therapeutic anticoagulation with coumadin, unfractionated heparin, enoxaparin (lovenox) and direct oral anticoagulants. (prophylactic anticoagulation is now standard of care and patients on prophylactic anticoagulation such as enoxaparin or unfractionated heparin, will be included in the study)
5. Vasodilatory shock.
6. Patient with known ongoing angina, recent myocardial infarction and sub-valvular aortic stenosis.
7. Active gastric or duodenal ulcer or any bleeding disorder or creatinine clearance < 10.

8. Hemoglobin <9 mg/dL, platelet count of <30,000 /mm^3.
9. Acute respiratory infection for >10 days.
10. Known allergy/hypersensitivity to Dipyridamole and/or Aspirin.
11. Severe hepatic or renal insufficiency.
12. Uncontrolled hypertension defined as systolic > 180 mm Hg or diastolic > 100 mm Hg.
13. Patients with known allergy to NSAIDs
14. Patients enrolled in other non-standard of care clinical trials.

4.2 Secondary Subjects

N/A

4.3 Number of Subjects

A. Total Number of Subjects

New Jersey Medical School, University Hospital in Newark will enroll approximately 132 subjects.

B. Total Number of Subjects If Multicenter Study

N/A.

C. Feasibility

Given the current incident rate, it is anticipated that enrollment will be completed within 6-8 months.

4.**4 Consent Procedures**

A. Consent Process

Provision of written or verbal Informed Consent will be obtained prior to any study-related procedures. The Investigator will ensure that the study participant is given full and adequate oral and written information about the nature, purpose, possible risk and benefit of the study. Study participants will also be notified that they are free to discontinue from the study at any time. The study participant will be given the opportunity to ask questions and allowed time to consider the information provided. The original Informed Consent Form will be given to the study participant.

Current FDA, OHRP, NIH, state and institutional regulations concerning informed consent will be followed. An IRB approved written informed consent document that embodies the elements of informed consent is required 45CFR46.116.

1. The investigator shall explain participation is voluntary and all aspects of the study, purpose of the study, treatment plan, procedures, risks, benefits, alternatives, and the right to refuse and withdraw at any time without penalty in lay language.
2. The investigator will answer all the patient’s questions regarding the study.
3. The investigator shall give the patient adequate opportunity to read the consent form and to discuss with family and friends.

4. If the patient/ authorized proxy decides to participate in the study, he/she will be asked to sign the Informed Consent Document. A copy of the signed Informed Consent Document will be given to the subject and this will be documented in the patient's medical record.
5. The investigator shall inform all patients they have the right to refuse study participation and withdraw their participation at any time without penalty and will be treated without prejudice.

- **Location of Consent Process**
  The consent form will be reviewed by the investigator, or a delegated study member, in the hospital room. Study participants must sign the consent in person at the participating institution. If the patient wishes to enroll on the study, he or she will sign the informed consent in the presence of a physician or nurse on the study team.
- **Ongoing Consent**
  If there are any changes to the protocol, these changes will have to be first reviewed and approved by the Rutgers IRB via an amendment before any change is implemented. Once approved, study participants will be notified of the protocol changes and will be provided with the updated informed consent form (ICF) for their signature, if necessary. Study participants will also be provided with a copy of the updated ICF for their records whenever re-consent is required.
- **Individual Roles for Researchers Involved in Consent**
  Only a treating investigator or research nurse listed within the IRB application will consent study participants to this study.
- **Consent Discussion Duration**
  It is anticipated that the informed consent process will take 30-60 minutes.
- **Coercion or Undue Influence**
  This study requires study participants to provide consent. Adults unable to consent due to their current condition in the absence of authorized proxy, will not be eligible for this study. The treating physician and/or research nurse will explain to the study participant that participation in this study is completely voluntary and the study participant can withdraw at any time.

▪ **Subject Understanding**

The treating investigators will seek consent only under circumstances that provide the prospective study participant the opportunity to consider whether to participate and that minimizes the possibility of coercion or undue influence. The information given to the patient will be in a language understandable to the patient. Sufficient time will be allowed for questions to be asked and answered, both by the patient and the individual obtaining consent to ensure the patient comprehends the consent information.

2. Waiver or Alteration of Consent Process

   N/A

3. Documentation of Consent

Documenting Consent

Written or telephone informed consent is required. Informed consent must be obtained prior to commencing any research procedures. Informed consent will be documented in accordance with 21CFR50.27; it shall be documented by the use of a written consent form approved by the

IRB and signed and dated by the study participant at the time of consent. A copy shall be given to the person signing the form.

▪Waiver of Documentation Of Consent (i.e., will not obtain subject's signature)
N/A

4.5 Special Consent/Populations

1. Minors-Subjects Who Are Not Yet Adults
   N/A

2. Wards of the State
   N/A

3. Non-English-Speaking Subjects

Both men and women and members of all ethnic groups are eligible for this trial. No special recruitment will be performed based on gender or minority status.
Where informed consent is documented in accordance with 46 CFR117 (b) (1), the written consent document should embody, in language understandable to the study participant, all the elements necessary for legally effective informed consent.

Process for Non-English-Speaking Subjects

1. An IRB approved short form written document shall be presented in a language understandable to the study participant.
2. The IRB approved English informed consent document may serve as a summary.
3. An interpreter fluent in English and the patient's language shall present the English IRB consent form offered, read it and orally present it to the patient.
4. Through the interpreter, the investigator will explain participation is voluntary and all aspects of the study, the purpose of the study, treatment plan, procedures, risks, benefits, alternatives, and the right to refuse and withdraw at any time without penalty in lay language.
5. Through the interpreter, the investigator will answer any questions the patient may have.
6. The investigator shall give the study participant adequate opportunity to take the IRB approved English informed consent document home for review by family and/or friends who are fluent in English and the language understandable to the patient.
7. If the patient decides to participate in the study, he/she will be asked to sign the short form document.
8. The IRB approved informed consent document will be signed by the person obtaining consent for authorized under the protocol.
9. The written short form document and the English informed consent document will be signed by the witness.
10. When the person obtaining consent is assisted by a translator, the translator may serve as a witness.
11. A copy of the signed IRB approved short form document and the English informed consent document will be given to the study participant and this will be documented in the patient's medical record.
12. Through the interpreter, the investigator shall inform all patients they have the right to refuse study participation and withdraw their participation at any time without penalty and will be treated without prejudice.

Short Form Consent for Non-English Speakers
Study participants who do not speak English will be presented with a short form consent document written in a language understandable to them.

C. Adults Unable to Consent / Decisionally Impaired Adults

Surrogate consent will be utilized if patients are not able to consent themselves. If and when patients are able to provide consent, they will be re-consented.

4.6 Economic Burden and/or Compensation for Subjects

1. Expenses

The study drugs will be provided at no charge by the Sponsor of the study. Study participants will not be charged for the cost of any research procedures which are conducted as part of this study. Patients and/or their insurance carriers (or Charity Care in the absence of insurance) will be expected to pay for the standard of care costs related to COVID-19 treatment and follow-up.

2. Compensation/Incentives

Study participants will not receive compensation for participation.

C. **Compensation Documentation**

N/A

4.7 Risks of Harm/Potential for Benefits to Subjects

A. Description of Risks of Harm to Subjects

Reasonably Foreseeable Risks of Harm (this data I got from Aggrenox product information available online- Appendix is attached for your review))

Risk of Bleeding

AGGRENOX increases the risk of bleeding.
Risk factors for bleeding include the use of other drugs that increase the risk of bleeding (e.g., anticoagulants, antiplatelet agents, heparin, anagrelide, fibrinolytic therapy, and chronic use of NSAIDs).

Intracranial Hemorrhage In European Stroke Prevention Study-2 (ESPS2), the annualized event rate for intracranial hemorrhage was 0.39%/year in the AGGRENOX group, 0.26%/year in the extended-release dipyridamole (ER-DP) group, 0.24%/year in the aspirin (ASA) group, and 0.29%/year in the placebo groups.

Gastrointestinal (GI) Side Effects
GI side effects include stomach pain, heartburn, nausea, vomiting, and gross GI bleeding. Although minor upper GI symptoms, such as dyspepsia, are common and can occur anytime during therapy, physicians should remain alert for signs of ulceration and bleeding, even in the

absence of previous GI symptoms. Inform patients about the signs and symptoms of GI side effects and what steps to take if they occur. In ESPS2, the annualized event rate for gastrointestinal bleeding was 2.97%/year in the AGGRENOX group, 1.58%/year in the extended-release dipyridamole group, 2.06%/year in the aspirin group, and 1.40%/year in the placebo groups.

Peptic Ulcer Disease
Avoid using aspirin in patients with a history of active peptic ulcer disease, which can cause gastric mucosal irritation and bleeding.

Alcohol Warning
Because AGGRENOX contains aspirin, counsel patients who consume three or more alcoholic drinks every day about the bleeding risks involved with chronic, heavy alcohol use while taking aspirin.

Renal Failure
Avoid aspirin in patients with severe renal failure (glomerular filtration rate less than 10 mL/minute) [see Use in Specific Populations.

Hepatic Insufficiency
Elevations of hepatic enzymes and hepatic failure have been reported in association with dipyridamole administration.

Coronary Artery Disease
Dipyridamole has a vasodilatory effect. Chest pain may be precipitated or aggravated in patients with underlying coronary artery disease who are receiving dipyridamole. For stroke or TIA patients for whom aspirin is indicated to prevent recurrent myocardial infarction (MI) or angina pectoris, the aspirin in this product may not provide adequate treatment for the cardiac indications.

Hypotension
Dipyridamole produces peripheral vasodilation, which can exacerbate pre-existing hypotension.

Stress Testing with Intravenous Dipyridamole and Other Adenosinergic Agents
Clinical experience suggests that patients being treated with AGGRENOX capsules who also require pharmacological stress testing with intravenous dipyridamole or other adenosinergic agents (e.g. adenosine, regadenoson) should interrupt AGGRENOX capsules for 48 hours prior to stress testing.

Intake of AGGRENOX capsules within 48 hours prior to stress testing with intravenous dipyridamole or other adenosinergic agents may increase the risk for cardiovascular side effects of these agents and may impair the sensitivity of the test.

Drug Interaction Study Information Obtained From Literature

Adenosinergic agents (e.g. adenosine, regadenoson)
Dipyridamole has been reported to increase the plasma levels and cardiovascular effects of adenosine. Adjustment of adenosine dosage may be necessary. Dipyridamole also increases the cardiovascular effects of regadenoson, an adenosine A2A-receptor agonist. The potential

risk of cardiovascular side effects with intravenous adenosinergic agents may be increased during the testing period when dipyridamole is not held 48 hours prior to stress testing.

Angiotensin Converting Enzyme (ACE) Inhibitors
Because of the indirect effect of aspirin on the renin-angiotensin conversion pathway, the hyponatremic and hypotensive effects of ACE inhibitors may be diminished by concomitant administration of aspirin.

Acetazolamide
Concurrent use of aspirin and acetazolamide can lead to high serum concentrations of acetazolamide (and toxicity) due to competition at the renal tubule for secretion.

Anticoagulants and Antiplatelets
Patients taking AGGRENOX in combination with anticoagulants, antiplatelets, or any substance impacting coagulation are at increased risk for bleeding. Aspirin can displace warfarin from protein binding sites, leading to prolongation of both the prothrombin time and the bleeding time. Aspirin can increase the anticoagulant activity of heparin, increasing bleeding risk.

Anagrelide
Patients taking aspirin in combination with anagrelide are at an increased risk of bleeding.

Anticonvulsants
Salicylic acid can displace protein-bound phenytoin and valproic acid, leading to a decrease in the total concentration of phenytoin and an increase in serum valproic acid levels.

Beta Blockers
The hypotensive effects of beta blockers may be diminished by the concomitant administration of aspirin due to inhibition of renal prostaglandins, leading to decreased renal blood flow and salt and fluid retention.

Cholinesterase Inhibitors
Dipyridamole may counteract the anticholinesterase effect of cholinesterase inhibitors, thereby potentially aggravating myasthenia gravis.

Diuretics
The effectiveness of diuretics in patients with underlying renal or cardiovascular disease may be diminished by the concomitant administration of aspirin due to inhibition of renal prostaglandins, leading to decreased renal blood flow and salt and fluid retention.

Methotrexate
Salicylate can inhibit renal clearance of methotrexate, leading to bone marrow toxicity, especially in the elderly or renal impaired.

Nonsteroidal Anti-Inflammatory Drugs (NSAIDs)
The concurrent use of aspirin with other NSAIDs may increase bleeding or lead to decreased renal function.

Oral Hypoglycemics
Moderate doses of aspirin may increase the effectiveness of oral hypoglycemic drugs, leading to hypoglycemia.

Uricosuric Agents (probenecid and sulfinpyrazone)
Salicylates antagonize the uricosuric action of uricosuric agents.

ADVERSE REACTIONS (please see Appendix C for further details)

Clinical Trials Experience

Table.1 Incidence of Adverse Events in ESPS2a

Individual Treatment Group
Body System/Preferred Term:

	AGGRENOX n (%/year)[b]	ER-DP Alone n (%/year)[b]	ASA Alone n (%/year)[b]	Placebo n (%/year)[b]
**Total Number of Patients**	1650	1654	1649	1649
**Central and Peripheral Nervous System Disorders**				
Headache	647 (28.25)	634 (27.91)	558 (22.10)	543 (22.29)
**Gastrointestinal System Disorders**				
Dyspepsia	303 (13.23)	288 (12.68)	299 (11.84)	275 (11.29)
Abdominal Pain	289 (12.62)	255 (11.22)	262 (10.38)	239 (9.81)
Nausea	264 (11.53)	254 (11.18)	210 (8.32)	232 (9.53)
Diarrhea	210 (9.17)	257 (11.31)	112 (4.44)	161 (6.61)
Vomiting	138 (6.03)	129 (5.68)	101 (4.00)	118 (4.84)
**Platelet, Bleeding and Clotting Disorders**				
Hemorrhage NOS	**52 (2.27)** 24	24 (1.06)	46 (1.82)	24 (0.99)

[a] Reported by ≥1%/year of patients during AGGRENOX treatment where the incidence was at least 1%/year greater than in those treated with placebo.
[b] Annual event rate per 100 pt-years = 100* number of subjects with event/subject-years.

Subject-years is defined as cumulative number of days on treatment divided by 365.25. Note: ER-DP = extended-release dipyridamole 200 mg; ASA = aspirin 25 mg. The dosage regimen for all treatment groups is BID. NOS = not otherwise specified. Discontinuation due to adverse events in ESPS2 was 25% for AGGRENOX, 25% for extended-release dipyridamole, 19% for aspirin, and 21% for placebo (refer to Table 2).

Table 2 Incidence of Adverse Events that Led to the Discontinuation of Treatment
a Treatment Groups

	AGGRENOX n (%/year)[b]	ER-DP Alone n (%/year)[b]	ASA Alone n (%/year)[b]	Placebo n (%/year)[b]
**Total Number of Patients**	1650	1654	1649	1649
Patients with at least one	417 (18.21)	419 (18.44)	318 (12.59)	352 (14.45)

Adverse Event that led to treatment discontinuation				
Headache	165 (7.20)	166 (7.31)	57 (2.26)	69 (2.83)
**Gastrointestinal System Disorders**				
Abdominal Pain	74 (3.23)	64 (2.82)	56 (2.22)	52 (2.13)
Nausea	91 (3.97)	95 (4.18)	51 (2.02)	53 (2.18)
Vomiting	53 (2.31)	52 (2.29)	28 (1.11)	24 (0.99)

[a]Reported by ≥1%/year of patients during AGGRENOX treatment where the incidence was at least 1%/year greater than in those treated with placebo.
[b] Annual event rate per 100 pt-years = 100* number of subjects with event/subject-years. Subject-years is defined as cumulative number of days on treatment divided

Risk of Harm from an Intervention on a Subject with an Existing Condition

- Renal Failure
  Avoid aspirin in patients with severe renal failure (glomerular filtration rate less than 10 mL/minute) [see Use in Specific Populations.

- Hepatic Insufficiency
  Elevations of hepatic enzymes and hepatic failure have been reported in association with dipyridamole administration.

- Coronary Artery Disease
  Dipyridamole has a vasodilatory effect. Chest pain may be precipitated or aggravated in patients with underlying coronary artery disease who are receiving dipyridamole. For stroke or TIA patients for whom aspirin is indicated to prevent recurrent myocardial infarction (MI) or angina pectoris, the aspirin in this product may not provide adequate treatment for the cardiac indications.

- Peptic Ulcer Disease
  Avoid using aspirin in patients with a history of active peptic ulcer disease, which can cause gastric mucosal irritation and bleeding.

- Other Foreseeable Risks of Harm
  One potential risk is loss of confidentiality. We have taken several steps to minimize the probability of this risk.

- Observation and Sensitive Information
  N/A

Procedures which Risk Harm to Embryo, Fetus, and/or Pregnant Subjects

Pregnancy

<u>Risk Summary</u>
Available data from published studies and postmarketing experience with AGGRENOX use during pregnancy have not identified a clear association between AGGRENOX use and major

birth defects, miscarriage, or adverse maternal or fetal outcomes (see Data). AGGRENOX contains low-dose aspirin which is an NSAID (see Clinical Considerations). In animal reproduction studies, there were adverse developmental effects with administration of aspirin in rats and rabbits at doses about 66 and 44 times, respectively, the human exposure at the maximum recommended daily dose of aspirin-dipyridamole. Reproduction studies with dipyridamole in mice, rabbits, and rats have revealed no evidence of harm to the fetus up to doses about 25 times the maximum recommended daily human dose of aspirin/dipyridamole. Nonclinical data are suggestive of a possible potentiation of aspirin-related fetal toxicity when combined with dipyridamole (see Data).

The estimated background risk of major birth defects and miscarriage for the indicated population is unknown. All pregnancies have a background risk of birth defect, loss, or other adverse outcomes. In the U.S. general population, the estimated background risk of major birth defects and miscarriage in clinically recognized pregnancies is 2-4 and 15-20%, respectively.

Clinical Considerations
Labor and Delivery
AGGRENOX, which contains dipyridamole and low-dose aspirin, increases the risk for bleeding.

Maternal use of high-dose aspirin can result in excessive blood loss at delivery, prolonged gestation, prolonged labor, intracranial hemorrhage in premature infants, low birth weight, stillbirth, and neonatal death. Data Human Data Published data from clinical trials, observational studies, case series, and case reports over several decades have not identified a clear association between aspirin-dipyridamole use in pregnancy and major birth defects, miscarriage, or adverse maternal or fetal outcomes. However, these studies cannot definitively establish the absence of any aspirin-dipyridamole associated risks. Methodological limitations of these studies include variability in the timing and dose of drug exposure (e.g., most exposures occurred beyond the first trimester) and the small sample sizes of individual studies.

Animal Data

Aspirin has been shown to be teratogenic in rats (spina bifida, exencephaly, microphthalmia and coelosomia) and rabbits (congested fetuses, agenesis of skull and upper jaw, generalized edema with malformation of the head, and diaphanous skin) at oral doses of 330 mg/kg/day and 110 mg/kg/day, respectively. These doses, which also resulted in a high resorption rate in rats (63% of implantations versus 5% in controls), are, on a mg/m basis, about 66 and 44 times, respectively, the dose of aspirin contained in the maximum recommended daily human dose of aspirin-dipyridamole. Reproduction studies with dipyridamole have been performed in mice, rabbits and rats at oral doses of up to 125 mg/kg, 40 mg/kg, and 1000 mg/kg, respectively (about 1½, 2, and 25 times the maximum recommended daily human oral dose, respectively, on a mg/m basis) and have revealed no evidence of harm to the fetus due to dipyridamole. When 330 mg aspirin/kg/day was combined with 75 mg dipyridamole/kg/day in the rat at doses about 66 and 2 times, respectively, the maximum recommended daily human dose, the resorption rate approached 100%.

Lactation

Risk Summary
Based on data from a clinical lactation study in breastfeeding women taking low-dose aspirin, the metabolite salicylic acid is present in human milk in low levels (see Data). Dipyridamole is

also present in human milk. There is no information on the effects of AGGRENOX or dipyridamole on the breastfed infant or on milk production. There is insufficient information to determine the effects of aspirin on the breastfed infant and no information on the effects of aspirin on milk production. The developmental and health benefits of breastfeeding should be considered along with the mother's clinical need for AGGRENOX and any potential adverse effects on the breastfed child from AGGRENOX or from the underlying maternal condition.

Data
A published clinical study involved six exclusively breastfeeding women at 1 to 8 months postpartum who were taking 81 mg aspirin daily. Milk samples were collected at steady state, at 0, 1, 2, 4, 8, 12, and 24 hours after taking a dose of aspirin. Aspirin was undetectable in human milk. Salicylic acid was present in milk at low levels (average concentration of 24 ng/mL). Based on an average milk consumption of 150 mL/kg/day, the calculated relative infant dose was 0.4%. No adverse effects on the breastfed infants were noted.

Geriatric Use

Of the total number of subjects in ESPS2, 61% were 65 and over, while 27% were 75 and over. No overall differences in safety or effectiveness were observed between these subjects and younger subjects, and other reported clinical experience has not identified differences in responses between the elderly and younger patients, but greater sensitivity of some older individuals cannot be ruled out.

Patients with Severe Hepatic or Severe Renal Dysfunction

AGGRENOX has not been studied in patients with hepatic or renal impairment. Avoid using aspirin containing products, such as AGGRENOX, in patients with severe hepatic or severe renal (glomerular filtration rate).

C. Risks of Harm to Non-Subjects

The patient will be given a mask immediately upon arrival and escorted to a private room with a closed door. When evaluating the patient during the visit, research personnel will wear appropriate personal protective equipment (PPE) as recommended by the CDC. This includes the following:

- Gown
- Gloves
- Eye/face protection (I.e., goggles, face shield)
- If available, National Institute for Occupational Safety and Health (NIOSH)-certified disposable N95 or better respirator. The CDC has stated that surgical (face) masks have been determined to be an acceptable alternative to N95 or better respirators, when the supply chain of respirators cannot meet the demand so long as procedures that are likely to generate respiratory aerosols are not being performed. **The use of a nasopharyngeal swab to collect viral load specimens during this study is not considered an aerosolizing procedure.

4. Assessment of Social Behavior Considerations

N/A

5. **Minimizing Risks of Harm**
▪Certificate of Confidentiality

N/A

▪Provisions to Protect the Privacy Interests of Subjects

The risk associated with breach of privacy and loss of confidentiality is low. All data is located in RedCap which is password-protected and limited to authorized users.

Every effort will be made to maintain patient confidentiality. Research and hospital records are confidential. Patient's name or any other personally identifying information will not be used in reports or publications resulting from this study. The Food and Drug Administration or other authorized agencies (e.g., qualified monitors from Rutgers), may review patients records and as required.

Patients will be required to sign a HIPAA authorization, providing permission to use identifiable health information to be used and/or disclosed for research purposes. Provisions for protecting the privacy interests of include:

- Ensuring that the conditions under which a procedure is performed or information is collected (e.g., physical locations, telephone contact, mail or email solicitations) afford protections against interactions with participants being witnessed, overheard or inadvertently intercepted or viewed.
- Limiting the information being collected to only the minimum amount of data necessary to accomplish the research purposes.

The research team will make all efforts to ensure confidentiality is maintained by:

- Protecting PHI against public viewing;
- Proper storage and disposal of documents that contain PHI;
- Safeguarding computer workstations and databases that access PHI.

F**. Potential Benefits to Subjects**

There may be direct benefit to the patient for participation in this study because the study participant will receive Aggrenox, which is FDA approved and Dipyridamole has been shown to have some benefit in a small series of COVID-19 patients in an unpublished study from China. Although this does not guarantee that each individual patient will benefit.

Data gathered from this study may allow us to identify an effective treatment for COVID-19 that could result in benefit to the patient or society as whole in the future.

5.0 Special Considerations

5.1 **Health Insurance Portability and Accountability Act (HIPAA)**

It is necessary that PHI be accessed to achieve the goals of this study. All research personnel are trained in HIPAA and routine protections of PHI will apply.

The Informed Consent Form will incorporate wording that complies with relevant data protection and privacy legislation. In accordance with the Health Information Portability and Accountability Act (HIPAA), the written Informed Consent Form must include a subject authorization to release medical information to a regulatory authority, or Institutional Review Board (IRB), to grant access to subject's medical information that includes all hospital records relevant to the study, including subjects' medical history.

The core elements included in the HIPAA authorization are:

- A description of the information to be used or disclosed that identifies the information in a specific and meaningful fashion;
- The name or other specific identification of the person(s), or class of persons, authorized to make the requested use or disclosure;
- The name or other specific identification of the person(s), or class of persons, to whom the covered entity may make the requested use or disclosure;
- A description of each purpose of the requested use or disclosure;
- An expiration date or an expiration event that relates to the individual or the purpose of the use or disclosure;
- Signature of the individual and date.

5.2 Family Educational Rights and Privacy Act (FERPA)

N/A

5.3 Code of Federal Regulations Title 45 Part 46 (Vulnerable Populations)

N/A

5.4 General Data Protection Regulation (GDPR)

N/A

5.5 NJ Access to Medical Research Act (Surrogate Consent)

N/A

6.0 Data Management Plan

6.1 Data Analysis

The sample size of 132 patients (66 patients in each arm) was estimated to provide 90% power to detect a relative between-group difference of 20% reduction in composite ordinal scale (from day 0 to day 15) with assumption of mean composite ordinal scale value of 4.5 ± 1.6 (yes) at baseline (day 0) and two-sided P value of 0.05.[20] For primary efficacy, we will do an intention-to-treat analysis and include all patients who have undergone randomization and will adjust for the

minimization variables. We will analyze continuous measures by means of linear regression and present as mean differences. For binary outcomes, we will present data as risk ratios from a binomial regression model.

6.2 Data Security

1. The Human Research Oversight Committee (HROC) is responsible for oversight of protocol progress, conduct, and compliance. The Rutgers Cancer Institute of New Jersey's Data Safety Monitoring Plan (DSMP) activities fall under the purview of HROC. In order to ensure the execution of the protocol with a minimum number of obstacles, the HROC focuses on the following objectives:

   - Monitor data and safety of CINJ investigator-initiated trials, including serious adverse events (SAE), enrollment progress, stopping rules, etc.
   - Review the safety of all trials conducted at CINJ.
   - Review enrollment to cooperative group and industry-sponsored studies.
   - Review study compliance through deviation reports of all trials, Quality Assurance (QA) audit reports of CINJ investigator-initiated trials and external monitoring reports of all trials.
   - Conduct response review for investigator-initiated trials.

6.3 Data and Safety Monitoring

A. Data/Safety Monitoring Plan

The Principal Investigators will be responsible for monitoring the data, assuring protocol compliance, and conducting the safety reviews at the specified frequency, which must be conducted at a minimum of once a month (including when re-approval of the protocol is sought). The Rutgers Cancer Institute of New Jersey's Human Research Oversight Committee (HROC) will be the DSMB of record. During the review process, the principal investigators (serving as monitor) will evaluate whether the study should continue unchanged, require modification/amendment, or close to enrollment. Either principal investigator, IRB or either Data or Safety Monitoring Committee have the authority to stop or suspend the study or require modifications at each institution. The PIs will be responsible for sharing this information in a timely fashion and coordinating appropriate parallel actions at the other institution.

An audit will be conducted following enrollment of the first two (2) or three (3) patients. Subsequent audits will occur dependent on the rate of enrollment. More frequent audits of patient data and study conduct will occur if necessary. Prior audit findings and/or situations that may arise during the study will determine the need for more frequent auditing. All audit findings will be discussed with the principal investigator and the Rutgers University IRB.

B. Data/Safety Monitoring Board Details

The Human Research Oversight Committee (HROC) is responsible for oversight of protocol progress, conduct, and compliance. The Rutgers Cancer Institute of New Jersey's Data Safety Monitoring Plan (DSMP) activities fall under the purview of HROC. In order to ensure the

execution of the protocol with a minimum number of obstacles, the HROC focuses on the following objectives:

- Monitor data and safety of CINJ investigator-initiated trials, including serious adverse events (SAE), enrollment progress, stopping rules, etc.
- Review the safety of all trials conducted at CINJ.
- Review enrollment to cooperative group and industry-sponsored studies.
- Review study compliance through deviation reports of all trials, Quality Assurance (QA) audit reports of CINJ investigator-initiated trials and external monitoring reports of all trials.
- Conduct response review for investigator-initiated trials.

6.4 **Reporting Results**

A. Individual Subjects' Results

Results of individual patient assessments will be shared with the individual patient as these are part of the medical record and impact medical care. These results are part of routine care (safety labs, routine radiology procedures, and physical examinations).

B. Aggregate Results

Aggregate research results will not be shared with the study participants.

C. Professional Reporting

The policies and procedures of Rutgers University's legal department (see: Investigator's Handbook) will govern publication of the trial. It is expected that the results of this trial will be submitted for publication in a timely manner following the conclusion. The PI and all co-authors prior to submission or use must review any abstract or manuscript.

D. Clinical Trials Registration, Results Reporting and Consent Posting

ClinicalTrials.Gov Registration and Data Reporting: Under the terms of the Food and Drug Administration Modernization Act (FDAMA) and the Food and Drug Administration Amendments Act (FDAA), the Sponsor of the trial is solely responsible for determining whether the trial and its results are subject to the requirements for submission to the Clinical Trials Data Bank, http://www.clinicaltrials.gov. Information posted will allow subjects to identify potentially appropriate trials for their disease conditions and pursue participation by calling a central contact number for further information on appropriate trial locations and trial site contact information.

This is a research study which prospectively assigns human participants to health related interventions. The study has been registered on clinicaltrials.gov per FDA Regulations and will be updated at least every 6 months.

6.5 Secondary Use of the Data

N/A

7.0 Research Repositories – Specimens and/or Data

Not applicable – all specimens will be used to address the study objectives. Any unused specimens will be discarded at the conclusion of the study.

8.0 Approvals/Authorizations

Approval will be obtained from the Rutgers University Biosafety Committee.

9.0 Bibliography

1. Cucinotta D, Vanelli M. WHO Declares COVID-19 a Pandemic. *Acta Biomed.* 2020;91(1):157-160.
2. Fauci AS, Lane HC, Redfield RR. Covid-19 - Navigating the Uncharted. *N Engl J Med.* 2020;382(13):1268-1269.
3. Weiss P, Murdoch DR. Clinical course and mortality risk of severe COVID-19. *Lancet.* 2020;395(10229):1014-1015.
4. Terpos E, Ntanasis-Stathopoulos I, Elalamy I, et al. Hematological findings and complications of COVID-19. *Am J Hematol.* 2020.
5. Thachil J, Tang N, Gando S, et al. ISTH interim guidance on recognition and management of coagulopathy in COVID-19. *J Thromb Haemost.* 2020;18(5):1023-1026.
6. Langer F, Kluge S, Klamroth R, Oldenburg J. Coagulopathy in COVID-19 and Its Implication for Safe and Efficacious Thromboprophylaxis. *Hamostaseologie.* 2020.
7. Thachil J, Tang N, Gando S, et al. Type and dose of heparin in Covid-19: Reply. *J Thromb Haemost.* 2020.
8. McPherson JA, Barringhaus KG, Bishop GG, et al. Adenosine A(2A) receptor stimulation reduces inflammation and neointimal growth in a murine carotid ligation model. *Arterioscler Thromb Vasc Biol.* 2001;21(5):791-796.
9. Ralevic V, Burnstock G. Receptors for purines and pyrimidines. *Pharmacol Rev.* 1998;50(3):413-492.
10. Wang C, Lin W, Playa H, Sun S, Cameron K, Buolamwini JK. Dipyridamole analogs as pharmacological inhibitors of equilibrative nucleoside transporters. Identification of novel potent and selective inhibitors of the adenosine transporter function of human equilibrative nucleoside transporter 4 (hENT4). *Biochem Pharmacol.* 2013;86(11):1531-1540.
11. Macatangay BJC, Jackson EK, Abebe KZ, et al. A Randomized, Placebo-Controlled, Pilot Clinical Trial of Dipyridamole to Decrease Hiv-Associated Chronic Inflammation. *J Infect Dis.* 2019.
12. Parkinson FE, Ferguson J, Zamzow CR, Xiong W. Gene expression for enzymes and transporters involved in regulating adenosine and inosine levels in rat forebrain neurons, astrocytes and C6 glioma cells. *J Neurosci Res.* 2006;84(4):801-808.

13. Boswell-Casteel RC, Hays FA. Equilibrative nucleoside transporters-A review. *Nucleosides Nucleotides Nucleic Acids.* 2017;36(1):7-30.
14. Chakrabarti S, Freedman JE. Dipyridamole, cerebrovascular disease, and the vasculature. *Vascul Pharmacol.* 2008;48(4-6):143-149.
15. Mustafa SJ. Effects of coronary vasodilator drugs on the uptake and release of adenosine in cardiac cells. *Biochem Pharmacol.* 1979;28(17):2617-2624.
16. Li Z LX, Huang Yi-Y et al. . FEP-based screening prompts drug repositioning against COVID-19. 2020.
17. Liu X LZ, Liu S et al. . Therapeutic effects of dipyridamole on COVID-19 patients with 2 coagulation dysfunction. 2020.
18. Adedeji AO, Sarafianos SG. Antiviral drugs specific for coronaviruses in preclinical development. *Curr Opin Virol.* 2014;8:45-53.
19. Fata-Hartley CL, Palmenberg AC. Dipyridamole reversibly inhibits mengovirus RNA replication. *J Virol.* 2005;79(17):11062-11070.
20. Kohler D, Streienberger A, Morote-Garcia JC, et al. Inhibition of Adenosine Kinase Attenuates Acute Lung Injury. *Crit Care Med.* 2016;44(4):e181-189.
21. Huang B, Chen Z, Geng L, et al. Mucosal Profiling of Pediatric-Onset Colitis and IBD Reveals Common Pathogenics and Therapeutic Pathways. *Cell.* 2019;179(5):1160-1176 e1124.
22. Insel PA, Murray F, Yokoyama U, et al. cAMP and Epac in the regulation of tissue fibrosis. *Br J Pharmacol.* 2012;166(2):447-456.
23. Weyrich AS, Skalabrin EJ, Kraiss LW. Targeting the inflammatory response in secondary stroke prevention: a role for combining aspirin and extended-release dipyridamole. *Am J Ther.* 2009;16(2):164-170.
24. International Severe Acute Respiratory and Emerging Infections Consortium (ISARIC) home page.
25. Cao B, Wang Y, Wen D, et al. A Trial of Lopinavir-Ritonavir in Adults Hospitalized with Severe Covid-19. *N Engl J Med.* 2020.
